# MASLD and sarcopenia research (2012–2025): a multi-database bibliometric analysis

**DOI:** 10.3389/fnut.2026.1834112

**Published:** 2026-06-12

**Authors:** Keyan Gao, Liwen Xun, Shalei Hao, Min An, Lingfeng Zuo, Jiayu Wang, Ying Wang, Junhua He

**Affiliations:** Department of Endocrinology, The Second Hospital of Shanxi Medical University, Taiyuan, Shanxi, China

**Keywords:** bibliometrics, liver–muscle axis, metabolic dysfunction–associated steatotic liver disease, precision nutrition, sarcopenia

## Abstract

**Background:**

MASLD and sarcopenia share common pathophysiological mechanisms, including insulin resistance and chronic inflammation, and their coexistence has been associated with increased risks of hepatic fibrosis, cardiovascular events, and all-cause mortality. Mapping research trends and nutrition-related implications along the liver–muscle axis may help inform precision nutrition strategies.

**Materials and methods:**

English-language original articles and reviews published from January 2012 to December 2025 were retrieved from the Web of Science Core Collection and Scopus databases. A total of 701 unique publications were included. Bibliometric analyses, including descriptive statistics, collaboration network analysis, co-citation analysis, and keyword burst detection, were performed using the R package bibliometrix, CiteSpace, and VOSviewer. BERTopic modeling was applied to identify latent semantic themes and their temporal evolution. Relevant prospective cohort studies from PubMed were also reviewed to provide contextual evidence on longitudinal associations between muscle-related parameters and MASLD outcomes.

**Results:**

The field grew at an average annual rate of 36.6%, with China, South Korea, and the United States forming the core collaborative network; South Korean authors showed particularly high productivity and influence. The research focus shifted from molecular mechanisms to clinical phenotyping, risk prediction, and intervention-related topics. BERTopic modeling identified three emerging themes: sarcopenia–fibrosis risk, lipid metabolism with lifestyle interventions, and cardiometabolic comorbidity management. The prospective cohort studies suggested that low muscle mass and impaired muscle function were associated with a higher risk of MASLD onset or progression (HR 1.18–7.96), consistent with previously proposed mechanisms, including lipotoxicity, systemic inflammation, and gut–liver–muscle axis dysregulation.

**Conclusion:**

Research on MASLD and sarcopenia has progressively shifted from descriptive and mechanistic studies toward early fibrosis risk prediction, lifestyle-based interventions, and integrated metabolic management. Standardized muscle assessment and further investigation of the liver–muscle axis are needed to support precision nutrition strategies.

## 1 Introduction

Metabolic dysfunction–associated steatotic liver disease (MASLD), formerly known as nonalcoholic fatty liver disease (NAFLD), is characterized by excessive lipid accumulation in hepatocytes and is closely linked to metabolic syndrome (1). With the global rise in obesity and type 2 diabetes mellitus, the burden of MASLD has continued to increase. MASLD currently affects approximately 38% of adults and 7–14% of children and adolescents, and its adult prevalence is projected to exceed 55% by 2040 (2). Rather than a single pathological entity, MASLD represents a disease spectrum ranging from simple steatosis to metabolic dysfunction–associated steatohepatitis (MASH), with potential progression to hepatic fibrosis, cirrhosis, and hepatocellular carcinoma (3). Beyond liver-related outcomes, MASLD is also associated with incident type 2 diabetes, chronic kidney disease, sarcopenia, and extrahepatic malignancies (4, 5).

Sarcopenia is a progressive disorder characterized by the loss of skeletal muscle mass and function (6). It is increasingly recognized not only as an age-related condition but also as an important prognostic factor in chronic liver disease (7–9). Previous studies have shown that sarcopenia predicts adverse outcomes in patients with cirrhosis and is associated with MASLD progression, advanced fibrosis, and liver-related complications (10, 11). MASLD and sarcopenia share several metabolic disturbances, including visceral adiposity and insulin resistance, suggesting a close pathophysiological connection (12, 13). Nutrition is a key modulator of both hepatic and skeletal muscle metabolism and may therefore represent a practical link between these two conditions. Dietary patterns such as the Mediterranean diet, together with adequate intake of high-quality protein and nutrients such as vitamin D, may help reduce hepatic inflammation and support muscle anabolism, thereby potentially attenuating disease progression (14, 15). In contrast, poor dietary patterns and gut microbiota dysbiosis may aggravate liver–muscle axis dysfunction, underscoring the potential role of precision nutrition in integrated management (16–18).

As research on MASLD and sarcopenia continues to expand, a systematic mapping of the knowledge structure and emerging directions is needed. Bibliometric analysis uses large-scale literature data to characterize publication trends, collaboration patterns, citation structures, and thematic evolution. Compared with single-database analyses, integrating multiple literature sources may improve coverage and reduce database-specific bias. However, no bibliometric study has specifically focused on the intersection of MASLD and sarcopenia. Therefore, this study integrated publications from the Web of Science Core Collection and Scopus to provide broad and representative coverage of the field. In addition, prospective studies from PubMed were screened and descriptively reviewed to provide clinical context for longitudinal associations between muscle-related parameters and MASLD outcomes. By combining multi-source bibliometric analysis with BERTopic modeling, this study aimed to characterize the thematic evolution, research hotspots, and emerging trends at the intersection of MASLD and sarcopenia, with particular attention to nutrition-related implications.

## 2 Materials and methods

### 2.1 Data sources and cleaning

The primary bibliometric data were obtained from two major academic databases: the Web of Science Core Collection (WOSCC) and Scopus. These databases were selected because of their broad coverage and complementary indexing scopes, which allowed a more comprehensive representation of the literature on MASLD and sarcopenia. PubMed was searched separately to identify prospective cohort studies that could provide additional clinical context for longitudinal associations between muscle-related parameters and MASLD outcomes. PubMed records were not merged into the main bibliometric dataset.

The search period was set from January 1, 2012, to December 31, 2025. The search was limited to English-language original articles and reviews. Boolean operators were used to combine MASLD-related and sarcopenia-related terms. In WOSCC, a topic search was conducted using the following strategy: TS = (“Non-alcoholic Fatty Liver Disease” OR “Nonalcoholic Fatty Liver Disease” OR “Metabolic Associated Fatty Liver Disease” OR “Metabolic-associated Fatty Liver Disease” OR “Metabolic Dysfunction-associated Steatotic Liver Disease” OR “Metabolic Dysfunction Associated Steatotic Liver Disease” OR “Metabolic Steatohepatitis” OR “Nonalcoholic Steatohepatitis” OR “Non-alcoholic Steatohepatitis” OR “NAFLD” OR “MAFLD” OR “MASLD” OR “MASH” OR “NASH”) AND (sarcopen* OR myopeni* OR dynaponi*). In Scopus, the same terms were applied to the Title, Abstract, and Keywords fields. The complete search strategy is provided in Supplementary Table 1.

**Figure 1:**
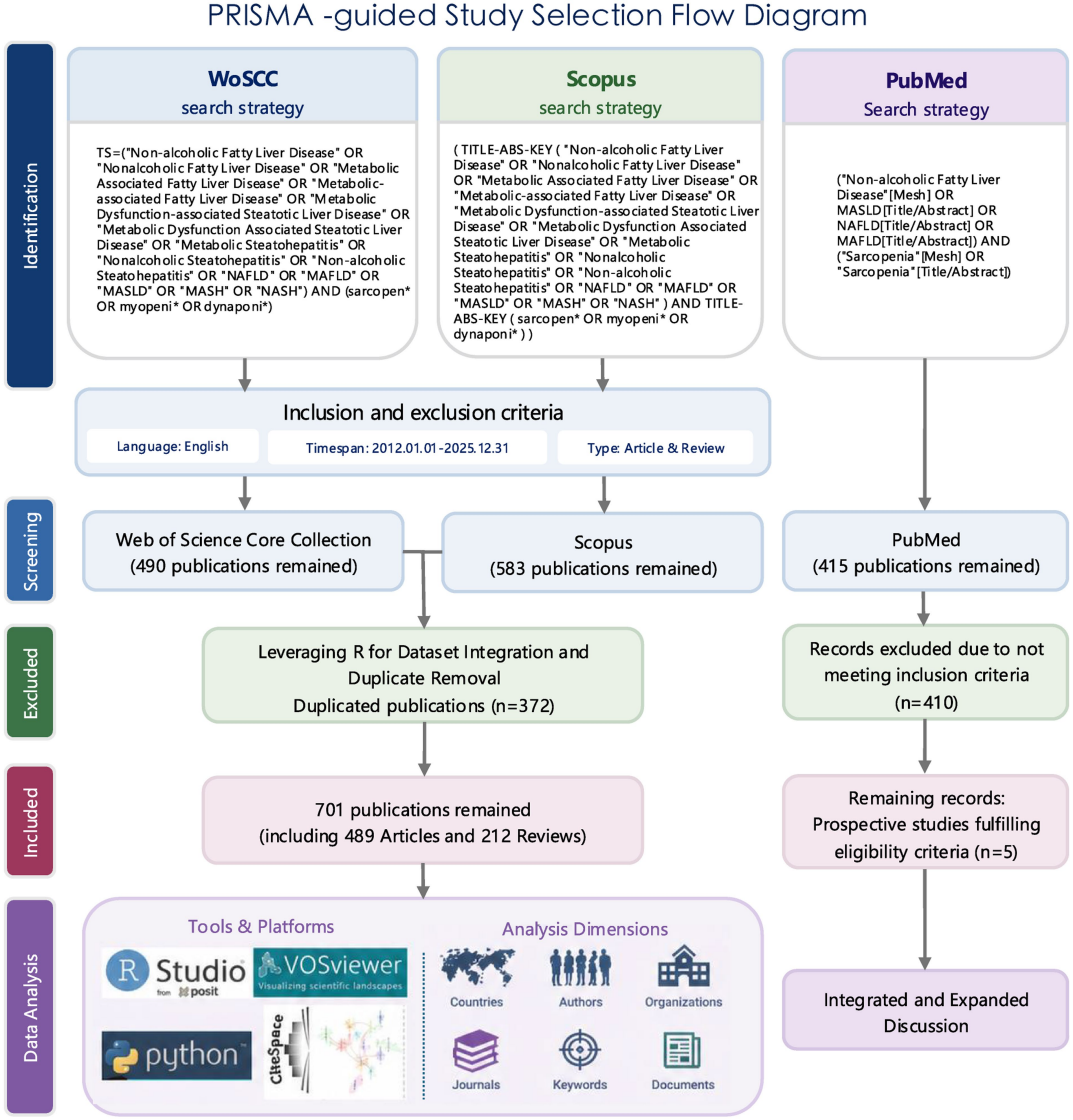
Literature screening and data integration (PRISMA flowchart). The workflow illustrates the number of records retrieved from the Web of Science Core Collection (WOSCC), Scopus, and PubMed databases using a comprehensive search strategy focused on “Non-alcoholic Fatty Liver Disease” (NAFLD), “Metabolic Dysfunction-associated Steatotic Liver Disease” (MASLD), and “Sarcopenia.” After removing duplicates and applying eligibility criteria (English language, Article and Review, 2012.01.01–2025.12.31), 701 unique publications were retained for bibliometric analysis. The integrated dataset was further processed using RStudio, VOSviewer, Python, and CiteSpace to analyze research trends across countries, authors, organizations, journals, keywords, and documents. Additionally, five prospective studies from PubMed were descriptively summarized to provide clinical context for the bibliometric findings.

The initial search yielded 490 records from WOSCC and 583 records from Scopus. Data integration and deduplication were performed using the R package bibliometrix and custom Python scripts (19). Duplicate records were identified by cross-referencing titles, authors, and publication years. After removing 372 duplicates, the final bibliometric dataset comprised 701 unique publications, including 489 original research articles and 212 reviews (Figure 1).

From the PubMed search, 415 records were screened, and five prospective cohort studies meeting the predefined eligibility criteria were selected for descriptive review (Supplementary Table 1; Figure 1). The inclusion criteria were as follows: (1) adult populations; (2) assessment of associations between sarcopenia or muscle-related parameters and MASLD; (3) prospective cohort or longitudinal study design; (4) MASLD or liver-related outcomes diagnosed using recognized methods, including imaging, scoring systems, clinical criteria, or disease codes; and (5) reported outcomes with extractable effect estimates, such as hazard ratios (HRs) with 95% confidence intervals (CIs). Exclusion criteria included non-original studies, such as reviews, editorials, conference abstracts, and case reports; cross-sectional studies; animal studies; studies without follow-up; and studies not assessing the relationship between sarcopenia-related parameters and MASLD outcomes. This descriptive review was intended to provide contextual clinical evidence rather than a formal systematic review or meta-analysis.

Titles, abstracts, and keywords from the included WOSCC and Scopus publications were used to construct the corpus for subsequent bibliometric and topic-modeling analyses. During data cleaning, the disambiguation functions of the R package bibliometrix were used to standardize bibliographic information. Text preprocessing included removal of non-alphabetic characters, punctuation, and redundant spaces to minimize formatting-induced noise.

### 2.2 Bibliometric analysis

A multidimensional bibliometric approach was used to analyze annual publication trends, core authors, journal distribution, citation structures, collaboration networks, and thematic evolution. Descriptive statistics and thematic analyses were performed using R software (version 4.4.5) with the bibliometrix package (version 5.0). Collaboration networks at the author, institution, and country levels were constructed using association-based normalization and Louvain clustering. Multiple correspondence analysis (MCA) was used to identify major keyword clusters. Theme centrality and thematic evolution analyses were based on high-frequency keywords to characterize structural relationships and temporal changes in research topics.

CiteSpace (version 6.2.R1) was used for keyword and citation burst detection. VOSviewer (version 1.6.20) was used for journal co-citation analysis to visualize inter-journal relationships and academic influence. Outputs from these tools were further processed and visualized in Python (version 3.12) using pandas, matplotlib, seaborn, and networkx (20–22).

### 2.3 BERTopic modeling

BERTopic was used to identify latent semantic topics and characterize their temporal dynamics, addressing the limitations of keyword-based bibliometric analyses in capturing contextual meaning. This method combines transformer-based document embeddings with dimensionality reduction and density-based clustering, allowing unsupervised extraction of semantically coherent topics without predefining the number of clusters.

Before modeling, texts were converted to lowercase, non-alphabetic characters were removed, and stopwords were excluded to reduce noise. Document embeddings were generated using the pre-trained SentenceTransformer model paraphrase-multilingual-MiniLM-L12-v2, which encoded each document into high-dimensional semantic vectors. UMAP was then applied for dimensionality reduction to preserve local semantic structure and improve cluster separation. HDBSCAN was subsequently used to identify dense regions and generate stable topic clusters. Representative keywords for each topic were extracted using class-based TF-IDF (c-TF-IDF), resulting in seven interpretable core topics.

Temporal dynamics were analyzed using the topics_over_time module. Linear regression was applied to assess changes in topic prevalence across publication years, with statistical significance set at *p* < 0.05. Topics spanning fewer than five years were excluded to improve temporal robustness. All topic-modeling analyses were conducted in Python using the BERTopic framework.

## 3 Results

### 3.1 General characteristics of publications

The integrated dataset from the Web of Science Core Collection (WOSCC) and Scopus included 701 unique publications published between 2012 and 2025 (Figure 1). These publications were authored by 4,027 researchers and appeared in 289 journals. The field showed substantial growth, with a compound annual growth rate (CAGR) of 36.57%. Original research articles accounted for the majority of publications (*n* = 489), while reviews also made a notable contribution (*n* = 212). The average number of authors per article was 7.34, and the international collaboration rate was 19.54% (Table 1; Supplementary Figure 1).

**Table 1:** Comparative overview of WOSCC, Scopus, and merged datasets.

Description	WOSCC	Scopus	WOSCC+Scopus
Timespan	2012:2025	2012:2025	2012:2025
Sources (journals, books, etc)	186	259	289
Documents	490	583	701
Annual growth rate %	32.15	35.21	36.57
Document average age	4.24	4.09	4.1
Average citations per doc	36.11	45.55	41.68
References	20,106	3,561	21,752
Keywords plus (ID)	973	3,922	3,185
Author’s keywords (DE)	860	1,006	1,161
Authors	2,880	3,226	4,027
Authors of single-authored docs	10	18	21
Single-authored docs	10	18	21
Co-authors per doc	7.5	7.15	7.34
International co-authorships %	18.57	20.93	19.54
Article	367	385	489
Review	123	198	212

Summary of dataset characteristics, including number of records retrieved, duplicates removed, and final unique publications used for analysis.

**Figure 2:**
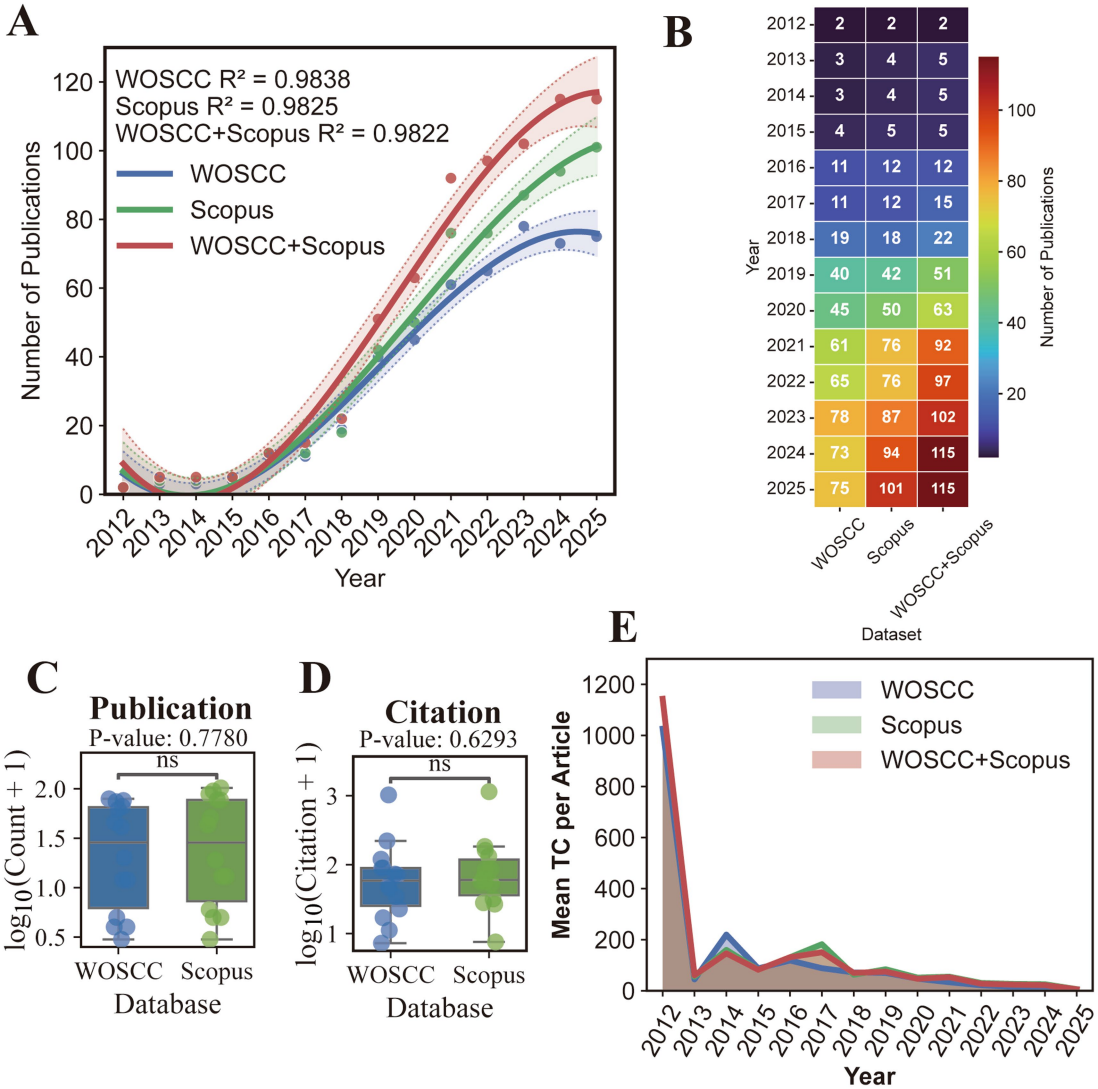
Temporal analysis of publication trends in MASLD and sarcopenia research. **(A)** Cubic polynomial regression curves fitted to the annual number of publications from 2012 to 2025, demonstrating high goodness-of-fit (R^2^ > 0.98) for WOSCC, Scopus, and combined datasets, indicating an accelerating growth in research output. **(B)** Heatmap visualization of publication counts across years and databases, highlighting the increasing research activity, particularly from 2019 onwards. **(C)** Boxplot comparison of publication counts (log10-transformed) between WOSCC and Scopus, showing no significant difference (*p* = 0.7780). **(D)** Boxplot comparison of citation counts (log10-transformed) between WOSCC and Scopus, also showing no significant difference (*p* = 0.6293). **(E)** Area plot depicting the mean total citations (TC) per article over time, reflecting the recency effect, as recent articles have had less time to accumulate citations.

Annual publication trends showed two distinct phases. From 2012 to 2016, the field remained in an exploratory phase, with annual output ranging from 2 to 12 publications. From 2018 onward, research activity expanded rapidly, increasing from 22 publications in 2018 to 115 in 2024. Polynomial trend fitting showed sustained growth across WOSCC, Scopus, and the combined dataset, with high goodness-of-fit (R^2^ > 0.98) (Figure 2A). Heatmap analysis further indicated increased research activity after 2019 (Figure 2B). Comparisons between WOSCC and Scopus showed no significant differences in annual publication counts (*p* = 0.7780) or citation distributions (*p* = 0.6293) (Figures 2C,D). Mean citations per article changed over time, reflecting the expected recency effect, as recently published articles had less time to accumulate citations (Figure 2E; Supplementary Table 2).

### 3.2 Publication trends and major contributors

**Figure 3:**
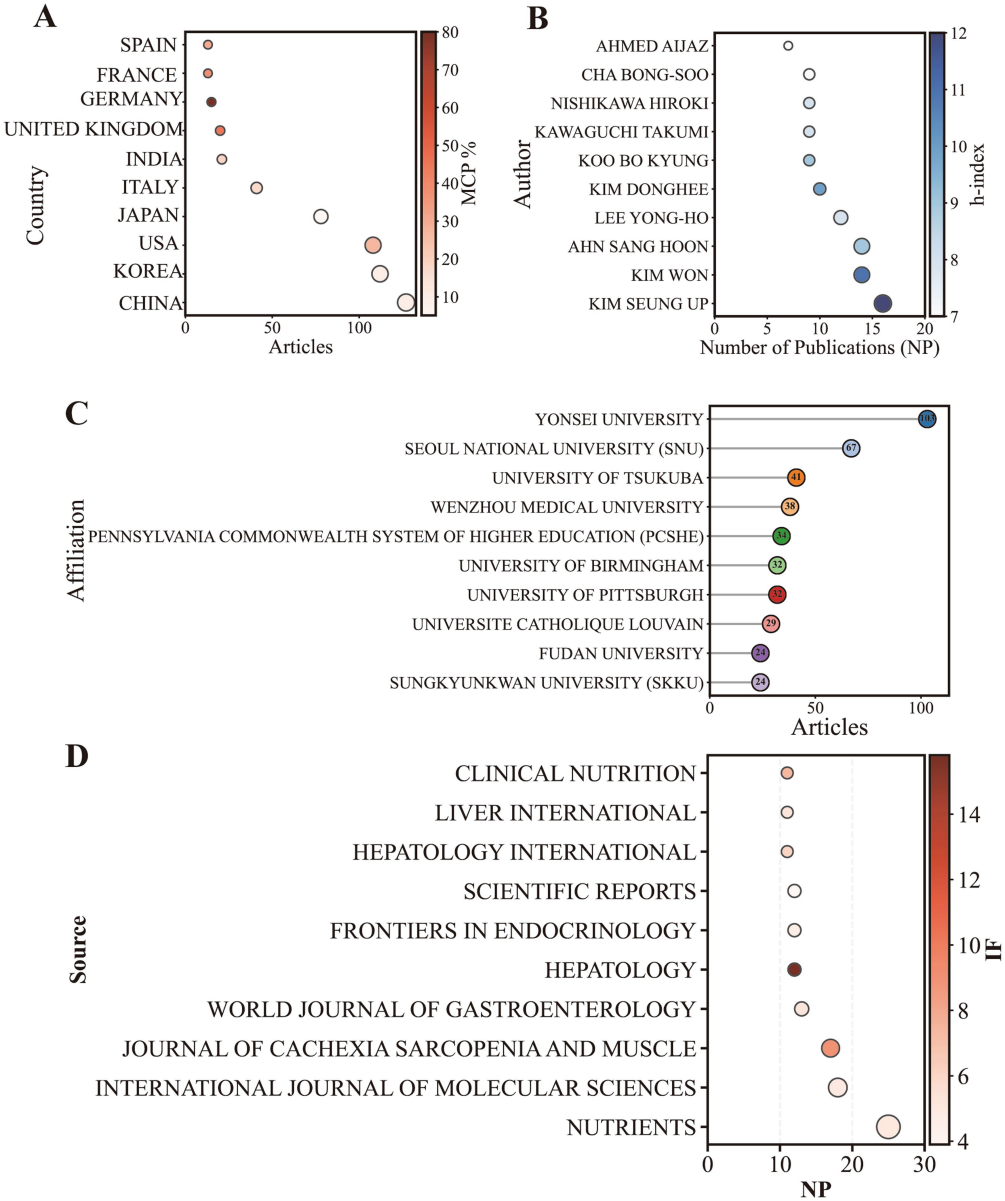
Global research output and collaboration network. **(A)** Country/region-level publication distribution and international collaboration rate (MCP%). The bubble size depicts the number of publications, while the color intensity indicates MCP%, reflecting the degree of international collaboration. China, Korea, and the USA show the highest publication outputs, with European countries (e.g., Spain, France, Germany) displaying notably high MCP%, indicating their central role in global research collaboration. **(B)** Core author productivity: top 10 authors by H-index, total citations, and number of publications (NP). The color gradient (blue scale) corresponds to H-index, with larger bubbles indicating higher publication counts. Authors such as Ahmed Aijaz, Cha Bong-Soo, and Nishikawa Hiroki demonstrate strong academic influence through high H-index and publication counts. **(C)** Leading research institutions by publication volume. The horizontal bar chart shows the top institutions contributing to MASLD and sarcopenia research, with Yonsei University (103 articles) and Seoul National University (67 articles) ranking highest. The color coding highlights institutional productivity, emphasizing the prominence of South Korean and Japanese universities in this field. **(D)** Journal distribution bubble plot. The *x*-axis represents the number of publications (NP), the *y*-axis shows total citations, and the bubble size corresponds to the H-index. Journals such as *Nutrients*, *Hepatology*, and *International Journal of Molecular Sciences* reflect high publication volumes and citation impact, demonstrating their key role in disseminating MASLD and sarcopenia research. The color gradient (red scale) further emphasizes the academic influence of these journals.

At the country level, China (127 publications, 18.1%), South Korea (112 publications, 16.0%), and the United States (108 publications, 15.4%) were the leading contributors, together accounting for nearly half of the global output. In contrast, several European countries had lower publication counts but higher international collaboration rates. Germany, the United Kingdom, and France contributed 15, 20, and 13 publications, respectively, with international collaboration rates of 80.0%, 45.0%, and 38.5%. These patterns suggest that European contributions were characterized more by cross-border collaboration than by publication volume alone (Figure 3A; Table 2).

Regarding author-level influence, South Korean scholars occupied a dominant position. Kim Seung Up ranked first, with an H-index of 12, 1,394 total citations, and 16 publications, followed by Kim Won (H-index = 11) and Ahn Sang Hoon (H-index = 9). Seven of the top 10 authors were from South Korea, forming a highly productive core author group. Kawaguchi Takumi from Japan entered the field relatively recently, beginning in 2021, but showed strong growth potential, as reflected by a high m-index of 1.333 (Figure 3B; Table 3).

**Table 2:** Top 10 countries by publication output and collaboration rate.

Country	Articles	Articles %	SCP	MCP	MCP %
China	127	18.1	114	13	10.2
Korea	112	16	101	11	9.8
USA	108	15.4	79	29	26.9
Japan	78	11.1	74	4	5.1
Italy	41	5.8	34	7	17.1
India	21	3	17	4	19
United Kingdom	20	2.9	11	9	45
Germany	15	2.1	3	12	80
France	13	1.9	8	5	38.5
Spain	13	1.9	9	4	30.8

Ranked by total publications (WOSCC + Scopus) and international collaboration proportion (MCP%), highlighting leading contributors and global collaboration patterns.

**Table 3:** Top 10 authors based on bibliometric indicators.

Author	h_index	g_index	m_index	TC	NP	PY_start
Kim Seung Up	12	16	1	1,394	16	2015
Kim Won	11	14	1.1	1,105	14	2017
Kim Donghee	10	10	1	802	10	2017
Ahn Sang Hoon	9	14	0.818	672	14	2016
Koo Bo Kyung	9	9	0.9	745	9	2017
Kawaguchi Takumi	8	9	1.333	867	9	2021
Lee Yong-Ho	8	12	0.667	1,237	12	2015
Nishikawa Hiroki	8	9	1.143	751	9	2020
Ahmed Aijaz	7	7	0.875	338	7	2019
Cha Bong-Soo	7	9	0.583	834	9	2015

This table lists H-index, total citations, and mean annual publications of the most prolific authors (WOSCC + Scopus), illustrating core research contributors.

Institutional output was concentrated in several East Asian centers. Yonsei University ranked first with 103 publications, followed by Seoul National University with 67 publications. Other major contributing institutions included the University of Tsukuba, Wenzhou Medical University, and Fudan University. Several North American and European institutions also appeared among influential contributors, indicating a geographically concentrated but internationally connected research landscape (Figure 3C).

Journal distribution reflected the interdisciplinary nature of the field. Journals such as *Nutrients*, *Hepatology*, and *International Journal of Molecular Sciences* showed high publication volumes or citation impact, suggesting that MASLD–sarcopenia research spans hepatology, nutrition, metabolism, and molecular medicine (Figure 3D; Table 4).

**Table 4:** Top 10 journals by bibliometric indicators.

Source	h_index	g_index	m_index	TC	NP	PY_start
Nutrients	14	25	1.4	653	25	2017
International Journal of Molecular Sciences	11	18	1.222	737	18	2018
Journal of Cachexia Sarcopenia and Muscle	10	17	0.667	1,029	17	2012
World Journal of Gastroenterology	8	13	0.615	440	13	2014
Hepatology	12	12	0.923	1984	12	2014
Frontiers in Endocrinology	7	12	1	263	12	2020
Scientific Reports	7	12	0.875	173	12	2019
Hepatology International	11	11	1.375	450	11	2019
Liver International	11	11	0.846	686	11	2014
Clinical Nutrition	9	11	0.9	1,329	11	2017

Ranked by total publications, total citations, and H-index (WOSCC + Scopus), reflecting journals with highest academic influence in MASLD and sarcopenia research.

### 3.3 Author productivity and core teams

**Figure 4:**
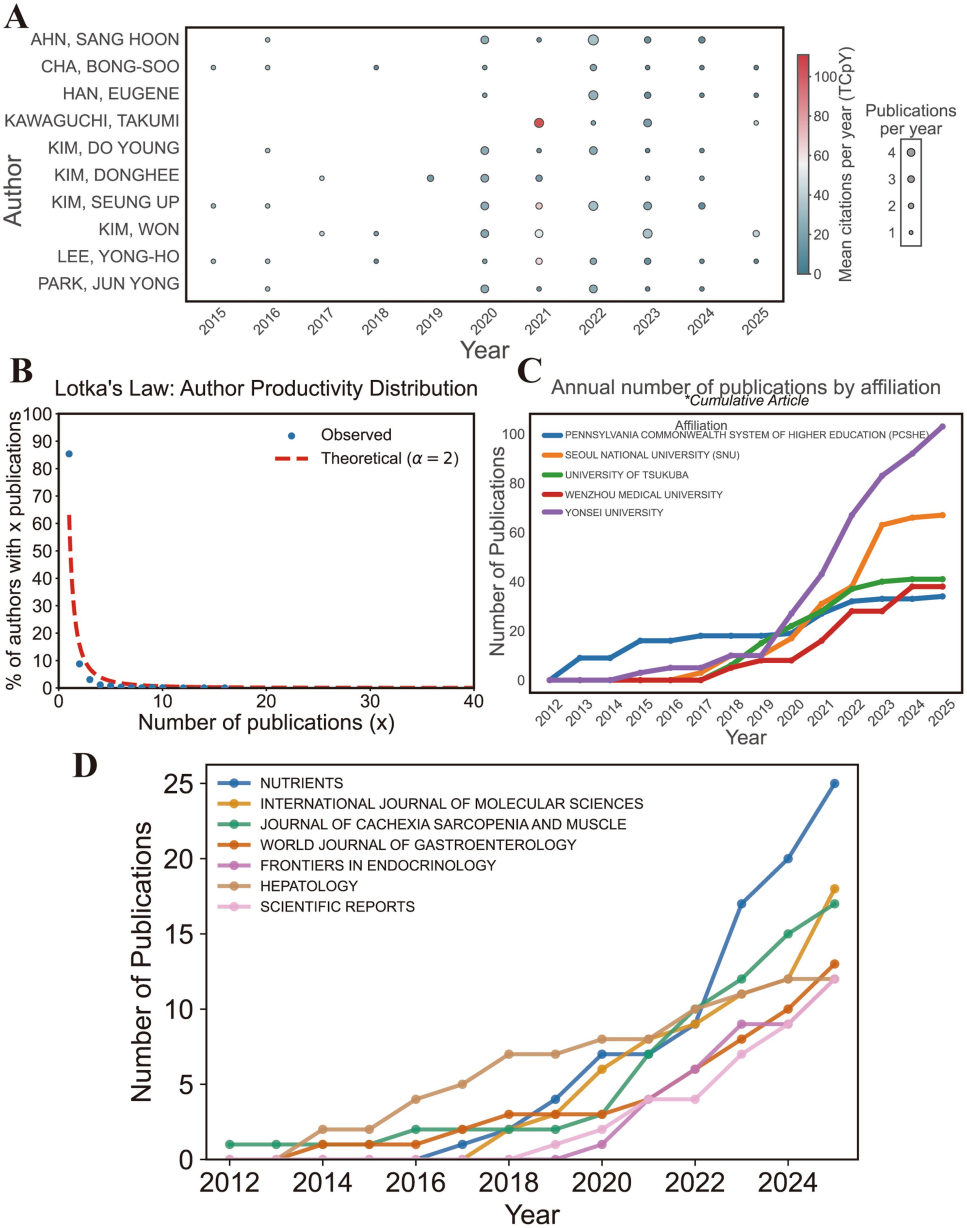
Temporal evolution of core authors, institutions, and journals. **(A)** Heatmap of annual publications by core authors. The bubble size represents the number of publications per year for each author, while the color gradient (from blue to red) indicates the mean citations per year (TCpY). Authors such as Kim, Do Young and Kim, Donghee show consistent productivity and high citation impact, particularly from 2020 onwards. **(B)** Author publication frequency distribution with Lotka’s law distribution fit. The observed distribution (blue dots) closely follows the theoretical Lotka’s law curve (red dashed line, *α* = 2). This confirms a “few prolific, many low-output” pattern in author productivity. This suggests that a small number of authors contribute significantly to the field, while the majority publish infrequently. **(C)** Time trajectories of cumulative publications by major institutions. The line plot shows the cumulative number of publications over time for top institutions. Yonsei University exhibits a marked growth advantage, with a steep increase in publications from 2020 onwards, surpassing other institutions such as Seoul National University (SNU) and the University of Tsukuba. **(D)** Annual publication trends of major journals. The line plot illustrates the number of publications per year in key journals. *Nutrients* and *International Journal of Molecular Sciences* show rapid growth in publication volume, reflecting a shift in focus from traditional hepatology journals (e.g., *Hepatology*) to nutrition and general medicine outlets. This trend highlights the interdisciplinary nature of MASLD and sarcopenia research.

Author-level analysis showed that publication output was concentrated among a relatively small group of prolific researchers, many of whom were based in South Korea. Lotka’s law fitting indicated that more than 85% of authors contributed only one publication, whereas core authors with two or more publications accounted for less than 15% of the total author pool (Figure 4B). This pattern suggests that the field remains driven by a limited number of active research groups. Some emerging contributors entered the core author network more recently but showed high citation impact per publication.

The heatmap of annual author productivity showed concentrated activity among core authors between 2020 and 2024 (Figure 4A). Cumulative publication trajectories further highlighted the rapid growth of Yonsei University and the steady output of Seoul National University and the University of Tsukuba (Figure 4C). In parallel, publication trends across major journals suggested a gradual shift from traditional hepatology outlets toward nutrition, metabolism, and general biomedical journals, reflecting the increasingly interdisciplinary nature of this research area (Figure 4D).

### 3.4 Collaboration networks

**Figure 5:**
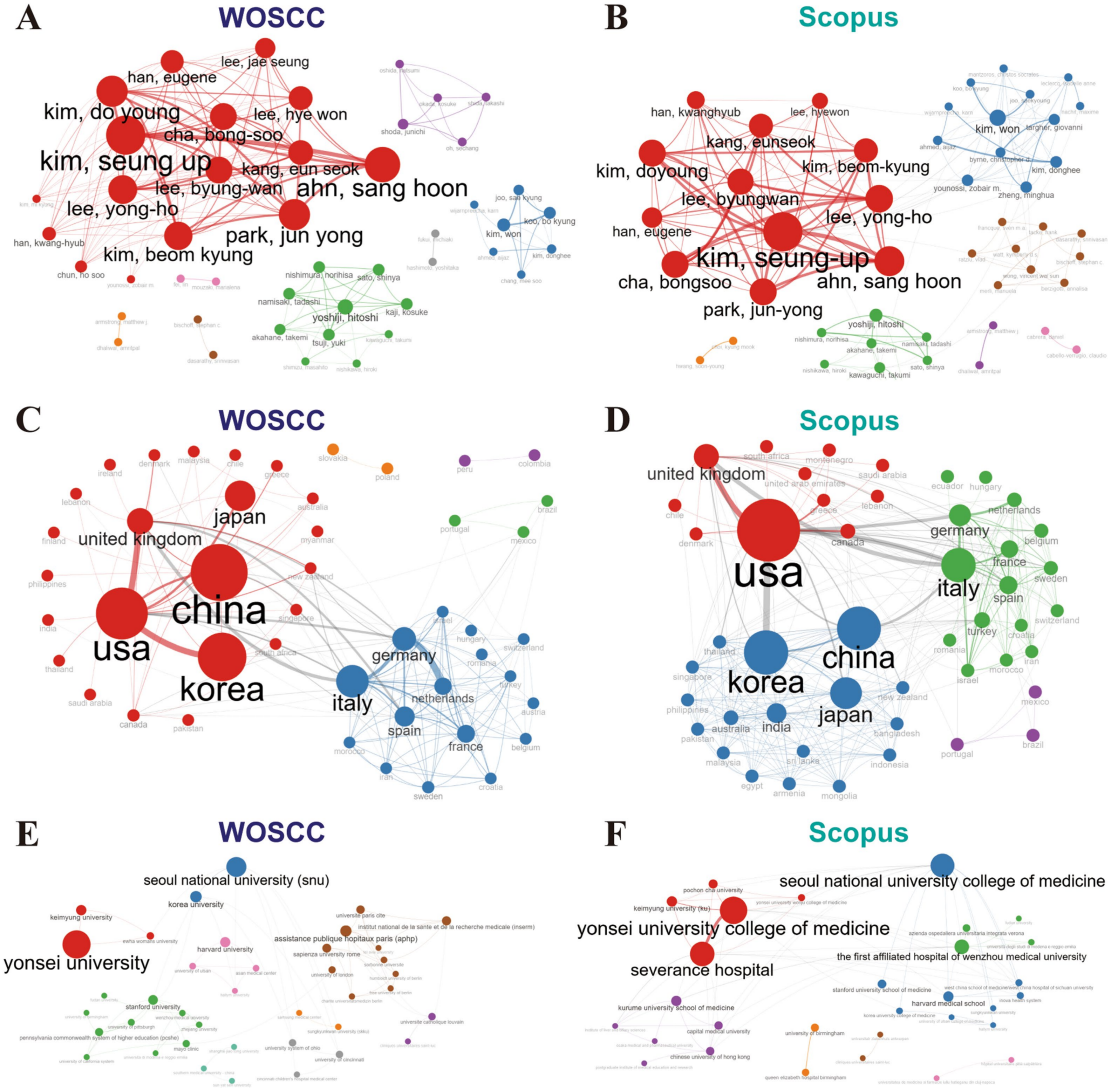
Visualization of collaborative networks at author, country, and institution levels. Collaboration networks were constructed using R-Bibliometrix to map the intellectual structure of the field. Node size is proportional to the number of publications, and edge thickness represents the strength of collaboration (co-authorship frequency). **(A,B)** Author collaboration networks in WOSCC and Scopus. These panels reveal distinct clusters of highly collaborative researchers. In both databases, a prominent “red cluster” emerges, centered on Korean scholars such as Kim Seung Up, Ahn Sang Hoon, and Lee Yong-Ho, illustrating a tightly knit research group driving significant output in this domain. The Scopus network **(B)** displays slightly more connections to international authors compared to the WOSCC network **(A)**, indicating broader global collaboration coverage in Scopus. **(C,D)** Country collaboration networks. The USA, China, and Korea appear as the largest nodes, signifying their dominance in publication volume. The USA acts as a central hub, showing strong collaborative links to the UK and China. Notably, European countries (e.g., Italy, Germany, Spain) form a dense sub-network, highlighting strong regional cooperation. The network topology in Scopus **(D)** shows a more interconnected global structure compared to WOSCC **(C)**. **(E,F)** Institutional collaboration networks. Yonsei University and Seoul National University (SNU) are identified as major hubs with the largest nodes, reflecting their leading roles in the field. The networks illustrate strong domestic collaboration within South Korea (e.g., between Yonsei and SNU) and emerging international links with institutions in the USA (e.g., Harvard University) and China (e.g., Wenzhou Medical University). The Scopus network **(F)** captures additional institutional nodes, providing a more granular view of the collaborative landscape.

Collaboration network analysis showed that East Asian researchers and institutions occupied central positions in the field. At the author level, both WOSCC and Scopus networks identified closely connected clusters centered on Korean scholars, including Kim Seung Up, Ahn Sang Hoon, and Lee Yong-Ho (Figures 5A,B). At the country level, China, South Korea, and the United States formed the main collaborative framework, while several European countries formed secondary clusters through regional collaborations and partnerships with the United States (Figures 5C,D).

Institutional networks further showed that Yonsei University and Seoul National University were major collaboration hubs. These institutions maintained strong domestic collaborations and had links with international institutions such as Harvard University and Wenzhou Medical University (Figures 5E,F). Overall, the collaboration structure suggests that several high-output East Asian groups play central roles, while broader international connections are developing across North America and Europe.

### 3.5 Keyword and thematic analysis

**Figure 6:**
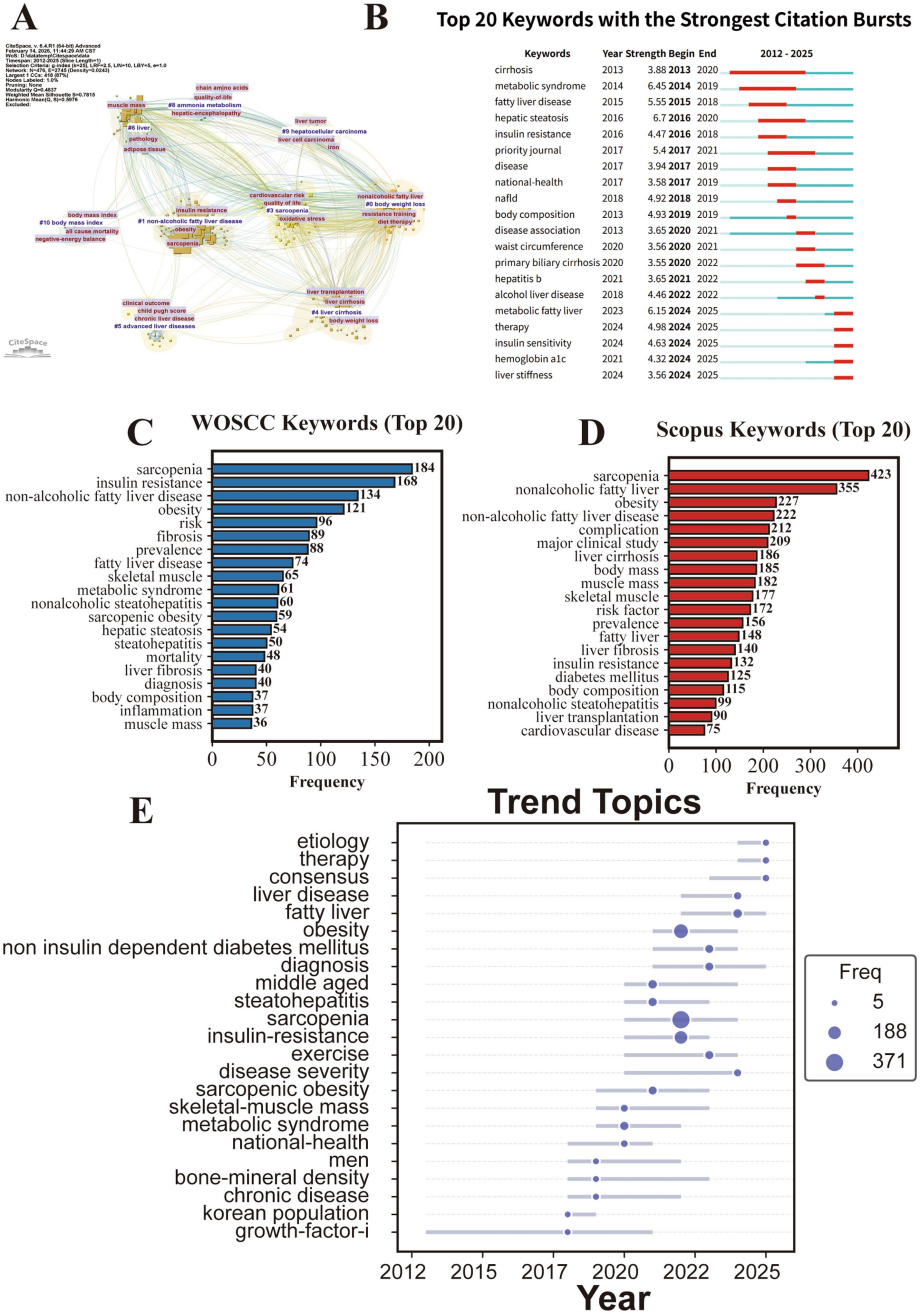
Keyword clustering, burst detection, and trend analysis. **(A)** Keyword co-occurrence network visualization. Generated using CiteSpace, this map illustrates the intellectual structure of the field. Nodes represent keywords, with size proportional to frequency. Links indicate co-occurrence relationships. The network is grouped into distinct clusters (e.g., #0 body weight loss, #1 non-alcoholic fatty liver disease, #3 sarcopenia), highlighting major research themes, including metabolic dysfunction, liver pathology, and muscle health interconnections. **(B)** Top 20 keywords with the strongest citation bursts. This table identifies keywords that experienced a sudden surge in citation frequency, indicating emerging research fronts. Early bursts (2013–2016) focus on “cirrhosis” and “metabolic syndrome,” while recent bursts (2024–2025) highlight “therapy,” “insulin sensitivity,” and “liver stiffness,” reflecting a shift toward clinical interventions and diagnostic precision. **(C,D)** Top 20 high-frequency keywords in WOSCC and Scopus. Bar charts compare keyword prevalence across databases. “Sarcopenia” and “insulin resistance” are dominant in WOSCC **(C)**, while Scopus **(D)** shows higher frequencies for “sarcopenia” and “nonalcoholic fatty liver.” Both databases confirm the centrality of metabolic and musculoskeletal terms, though Scopus captures a broader range of clinical terms like “obesity” and “complication.” **(E)** Trend topics over time. This timeline plot tracks the evolution of keyword usage from 2012 to 2025. Early research (2012–2015) focused on “etiology” and “diagnosis.” Recent years (2020–2025) show a surge in “sarcopenic obesity,” “skeletal-muscle mass,” and “growth-factor-I,” indicating a growing interest in the intersection of muscle wasting and metabolic liver disease.

Keyword analysis identified “sarcopenia” (*n* = 335) and “Non-alcoholic fatty liver disease” (*n* = 284; reflecting the historical terminology before MASLD) as the most frequent terms, followed by “insulin resistance” (*n* = 214) and “obesity” (*n* = 202) (Figures 6C,D; Supplementary Table 3). These keywords indicate that the field has been centered on the interaction between muscle loss, hepatic steatosis and metabolic dysfunction.

High-frequency clinical and phenotypic terms, including “Sarcopenic obesity” (*n* = 80) and “Body composition” (*n* = 71), suggest growing attention to muscle–fat interactions and body composition assessment. The keyword co-occurrence network showed close links among sarcopenia, NAFLD/MASLD, obesity, insulin resistance, and fibrosis-related terms (Figure 6A). Burst detection revealed a temporal shift in research priorities. Early studies from 2013 to 2018 focused mainly on foundational associations, including “Cirrhosis,” “Metabolic syndrome,” and “Hepatic steatosis.” From 2019 to 2022, research attention shifted toward refined phenotypes such as “Body composition” and “Waist circumference.” More recent bursts from 2023 to 2025 included “Metabolic fatty liver,” “Therapy,” “Insulin sensitivity,” and “Liver stiffness,” suggesting increasing interest in non-invasive assessment, metabolic characterization, and intervention-related topics (Figure 6B). Topic trend analysis also indicated a gradual shift from basic disease characterization toward clinical phenotyping, sarcopenic obesity, skeletal muscle mass, and lifestyle-related research directions (Figure 6E).

### 3.6 Citation analysis

**Figure 7:**
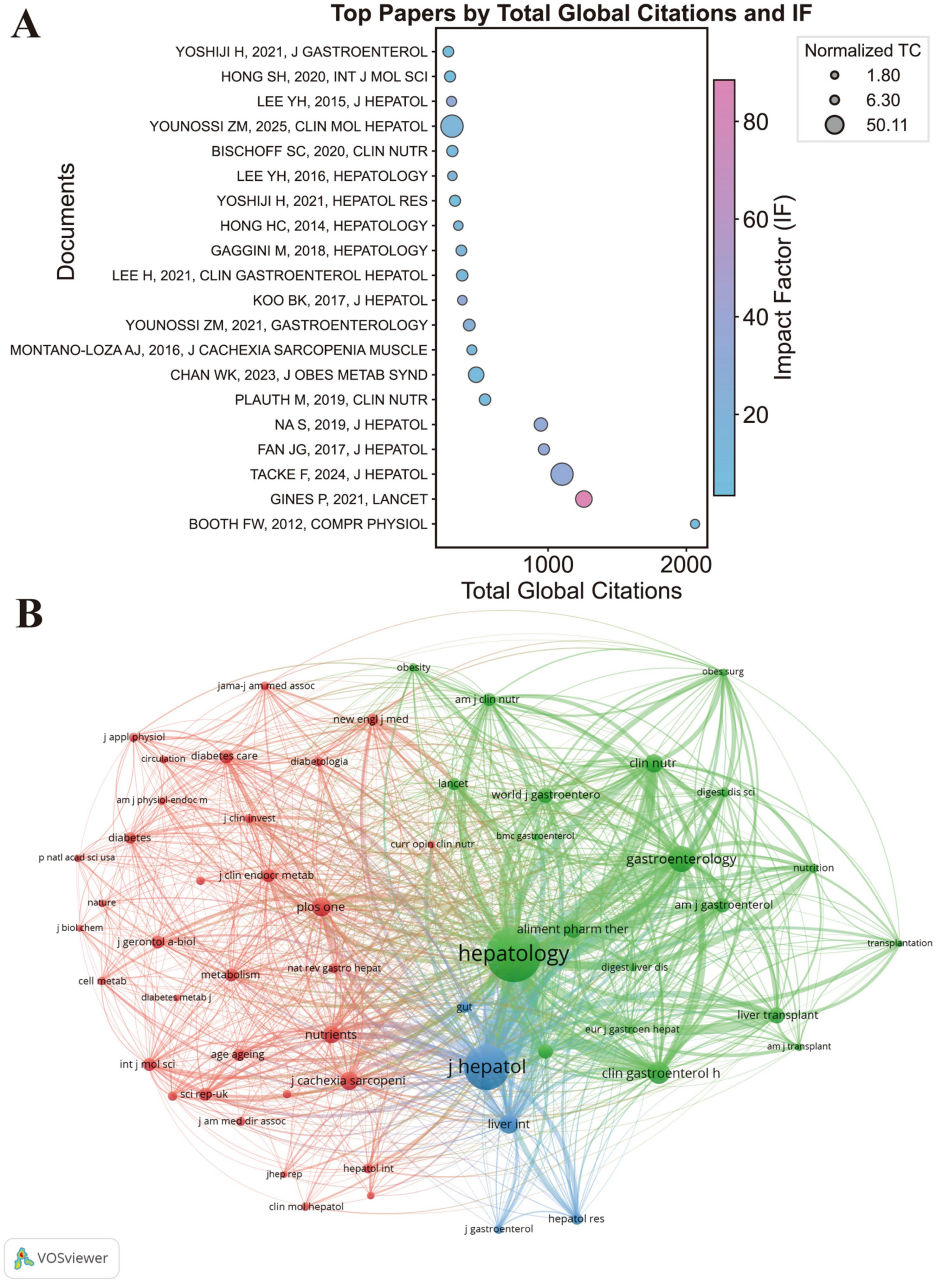
Citation analysis and journal co-citation network. **(A)** High-citation publications visualized as a bubble plot. This plot evaluates the academic impact of the top 20 most cited documents. The *x*-axis represents total global citations, indicating the overall influence, while the *y*-axis lists the specific documents (first author, year, journal). The color scale represents the impact factor (IF) of the publishing journal (ranging from blue to pink), and the bubble size corresponds to the normalized total citation score. **(B)** Journal co-citation network. Constructed using VOSviewer, this network map visualizes the intellectual structure of the field based on journal co-citation patterns. Node size reflects the number of co-citations, and line thickness indicates the strength of the relationship. The network is divided into three distinct color-coded clusters: green cluster (Hepatology and Gastroenterology): centered around “hepatology” and “gastroenterology,” this is the largest cluster, representing the core medical domain of the research. It includes specialized journals like Am J Gastroenterol and Liver Transplant. Red Cluster (Metabolism and General Medicine): This cluster links liver disease with broader metabolic and physiological contexts. Key nodes include “diabetes,” “obesity,” and high-impact general journals like Nature and New Engl J Med. Blue Cluster (Clinical Nutrition and Intervention): centered on “J Hepatol” and “Clin Nutr,” this cluster highlights the intersection of liver disease management and nutritional intervention. Synthesis: The central position of “hepatology” and its strong links to the nutrition cluster suggest that while the research is rooted in liver specialty, it heavily relies on nutritional science for therapeutic strategies.

Citation analysis identified both foundational and recently highly cited publications that shaped scholarly attention in the field. Publications by Booth FW (2012) and more recent studies by Tacke *F* (2024) and Younossi ZM (2025) were among the major citation nodes (Figure 7A; Table 5).

**Table 5:** Top 20 most cited publications.

Paper	DOI	Total citations	TC per year	Normalized TC
Booth FW, 2012, Compr Physiol	https://doi.org/10.1002/cphy.c110025	2063	137.53	1.80
Giǹes P, 2021, Lancet	https://doi.org/10.1016/S0140-6736(21)01374-X	1,259	209.83	23.66
Tacke F, 2024, J. Hepatol.	https://doi.org/10.1016/j.jhep.2024.04.031	1,101	367.00	49.61
Fan J-G, 2017, J. Hepatol.	https://doi.org/10.1016/j.jhep.2017.06.003	970	97.00	6.43
NA, 2019, J Hepatol	https://doi.org/10.1016/j.jhep.2018.06.024	948	118.50	12.63
Plauth M, 2019, Clin Nutr	https://doi.org/10.1016/j.clnu.2018.12.022	544	68.00	7.25
Chan W-K, 2023, J. Obe. Met. Synd.	https://doi.org/10.7570/jomes23052	480	120.00	19.83
Montano-Loza AJ, 2016, J Cachexia Sarcopeni	https://doi.org/10.1002/jcsm.12039	449	40.82	3.41
Younossi ZM, 2021, Gastroenterology	https://doi.org/10.1053/j.gastro.2020.11.051	430	71.67	8.08
Koo BK, 2017, J Hepatol	https://doi.org/10.1016/j.jhep.2016.08.019	380	38.00	2.52
Lee H, 2021, Clin Gastroenterol H	https://doi.org/10.1016/j.cgh.2020.12.022	379	63.17	7.12
Gaggini M, 2018, Hepatology	https://doi.org/10.1002/hep.29465	373	41.44	5.33
Hong HC, 2014, Hepatology	https://doi.org/10.1002/hep.26716	351	27.00	2.39
Yoshiji H, 2021, Hepatol Res	https://doi.org/10.1111/hepr.13678	327	54.50	6.14
Lee YH, 2016, Hepatology	https://doi.org/10.1002/hep.28376	308	28.00	2.34
Bischoff SC, 2020, Clin Nutr	https://doi.org/10.1016/j.clnu.2020.09.001	308	44.00	6.54
Younossi ZM, 2025, Clin Mol Hepatol	https://doi.org/10.3350/cmh.2024.0431	305	152.50	50.11
Lee YH, 2015, J Hepatol	https://doi.org/10.1016/j.jhep.2015.02.051	302	25.17	3.66
Hong SH, 2020, Int J Mol Sci	https://doi.org/10.3390/ijms21020494	291	41.57	6.18
Yoshiji H, 2021, J Gastroenterol	https://doi.org/10.1007/s00535-021-01788-x	279	46.50	5.24

Details include authors, title, publication year, journal, total citations, and citation dynamics (WOSCC + Scopus).

Journal co-citation analysis showed that *Hepatology* and *Journal of Hepatology* occupied central positions, while nutrition, metabolism, and clinical journals contributed to cross-disciplinary integration (Figure 7B; Table 6). The dual-map overlay further illustrated citation flows across clinical medicine, molecular biology, nutrition, and systems-related domains, highlighting the multidisciplinary structure of MASLD–sarcopenia research (Supplementary Figure 2). Citation burst analysis further highlighted references that received rapid increases in attention over specific periods, reflecting shifts in scholarly focus over time (Supplementary Figure 3).

**Table 6:** Top 20 co-cited journals.

Source	Citations	Total link strength
Hepatology	2,314	134,986
J Hepatol	1757	104,686
Gastroenterology	808	66,637
Clin Gastroenterol H	630	47,148
Liver Int	518	36,642
Plos One	517	29,963
Clin Nutr	503	48,714
J Cachexia Sarcopeni	487	25,349
Nutrients	466	30,833
Aliment Pharm Ther	450	32,508
Liver Transplant	414	29,386
World J Gastroentero	375	27,923
J Gastroen Hepatol	358	24,357
Am J Gastroenterol	347	32,318
Diabetes Care	318	19,321
Metabolism	315	17,630
J Clin Endocr Metab	313	19,182
Am J Clin Nutr	312	29,149
Int J Mol Sci	309	17,567
J Gerontol A-Biol	297	13,008

Ranked by co-citation frequency, highlighting journals forming central hubs and bridging roles in the knowledge network.

### 3.7 Thematic and topic evolution

**Figure 8:**
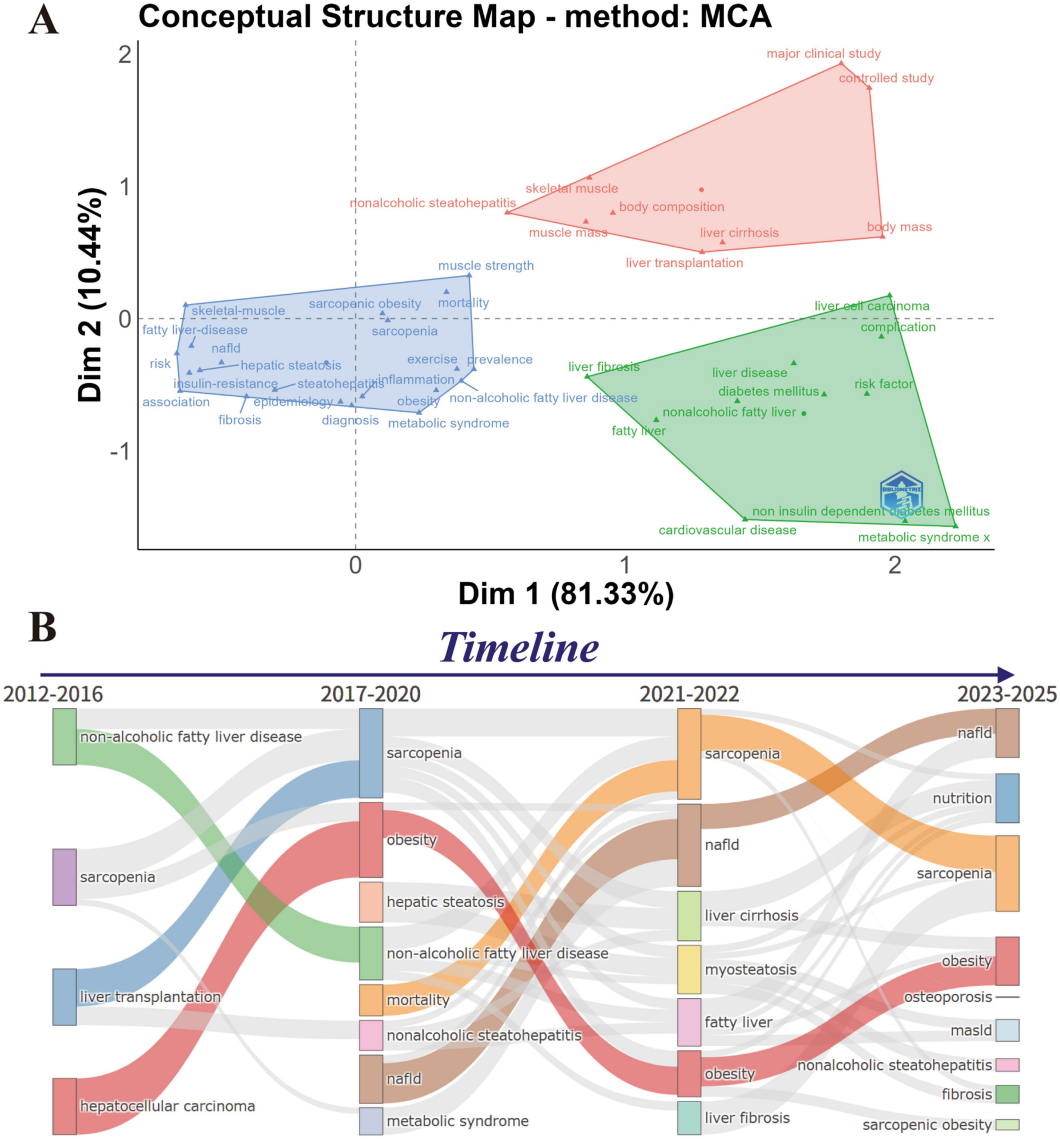
Thematic evolution based on multiple correspondence analysis (MCA). **(A)** MCA conceptual structure. This map depicts the intellectual landscape derived from keyword co-occurrence using MCA. Dimension 1 (81.33% of variance) separates the field into three principal clusters: (i) Clinical and physiological assessment (e.g., major clinical study, body composition, skeletal muscle, liver transplantation), reflecting patient phenotyping and surgical outcomes; (ii) pathophysiological mechanisms, centered on sarcopenia, insulin resistance, and non-alcoholic fatty liver disease (NAFLD), highlighting metabolic and hepatic interplay; (iii) disease progression and comorbidities, linking liver fibrosis, diabetes mellitus, hepatocellular carcinoma, and cardiovascular disease, indicating a focus on long-term complications and risk stratification. **(B)** Theme evolution Sankey diagram (2012–2025). This diagram illustrates the temporal evolution of research themes across four phases, reflecting shifts in scholarly focus rather than clinical causality. 2012–2016 (foundational phase): Research centered on NAFLD and liver transplantation, with emerging interest in sarcopenia and hepatocellular carcinoma. 2017–2020 (intersection phase): Increasing integration of hepatology and body composition, with emphasis on sarcopenia, obesity, and mortality as prognostic factors. 2021–2022 (refinement phase): Growing specialization, exemplified by myosteatosis, and closer integration of sarcopenia and obesity within NAFLD research. 2023–2025 (current frontiers): Transition toward MASLD terminology, with emerging hotspots including nutrition, sarcopenic obesity, and osteoporosis, reflecting a shift toward a multisystem perspective in metabolic liver disease.

Multiple correspondence analysis (MCA), BERTopic modeling, and Sankey-based thematic evolution analysis revealed the temporal development of research themes (Figure 8). In the MCA plot, Dimension 1 explained 81.33% of the variance and separated three major thematic clusters: clinical and physiological assessment, pathophysiological mechanisms, and disease progression with comorbidities. These clusters included terms related to body composition, skeletal muscle, insulin resistance, NAFLD/MASLD, fibrosis, diabetes mellitus, hepatocellular carcinoma, and cardiovascular disease (Figure 8A).

The Sankey-based thematic evolution diagram showed four broad developmental stages from 2012 to 2025. During the foundational phase from 2012 to 2016, research centered mainly on NAFLD, liver transplantation, sarcopenia, and hepatocellular carcinoma. From 2017 to 2020, research increasingly linked hepatology with body composition assessment, with emphasis on sarcopenia, obesity, and mortality. During the refinement phase from 2021 to 2022, myosteatosis and the overlap between sarcopenia and obesity became more prominent. In the most recent phase from 2023 to 2025, the field shifted toward MASLD terminology, with emerging attention to nutrition, sarcopenic obesity, and osteoporosis. These thematic changes reflect shifts in scholarly focus rather than evidence of causal relationships or direct clinical effects (Figure 8B).

### 3.8 BERTopic-based topic trends

**Figure 9:**
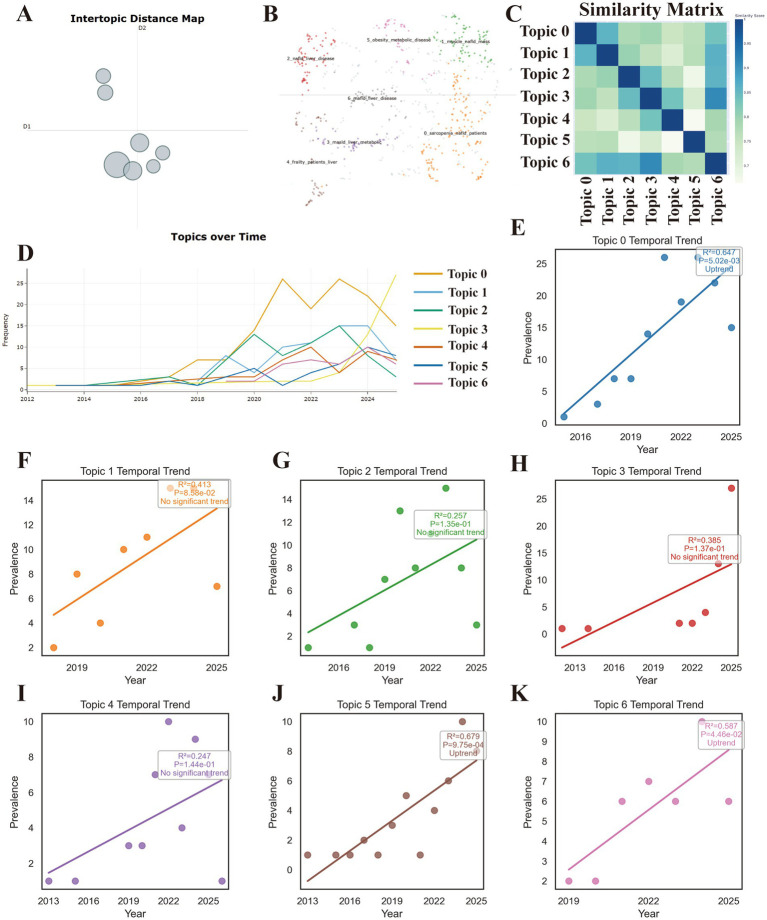
Latent topic identification and temporal trend analysis using BERTopic. This analysis employs a transformer-based topic modeling approach to uncover hidden semantic structures within the corpus and track their evolution over time. **(A–C)** Topic distance map, cluster scatter plot, and similarity matrix. These panels visualize the dimensionality reduction and clustering of the document corpus, segmenting the literature into seven distinct semantic topics (Topic 0–6). The topic distance map **(A)** illustrates the inter-topic relationships based on c-TF-IDF representations, where the spatial distance reflects the dissimilarity of keyword distributions. The clear separation observed in the cluster scatter plot **(B)** and the block-like patterns in the similarity matrix **(C)** validate the robustness of these thematic groupings. **(D)** Annual frequency trends per topic. This panel illustrates the publication volume trajectory for each identified topic, revealing a notable exponential growth in research output across the board post-2018, which indicates a rapid expansion and intensification of interest in this field. **(E–K)** Linear regression of topic popularity over time. These plots quantify the temporal evolution of specific research themes. The analysis highlights three core, rapidly growing topics: Topic 0 (focused on sarcopenia and fibrosis risk), Topic 5 (centered on lipid metabolism and lifestyle intervention), and Topic 6 (addressing MAFLD and cardiometabolic risk). The annotated R^2^ and *p*-values confirm the statistical significance of these upward trends, identifying them as the dominant frontiers in current hepatology research.

BERTopic modeling identified seven interpretable core topics, numbered from 0 to 6. The topic distance map, cluster scatter plot, and similarity matrix showed distinct semantic groupings within the corpus, supporting the interpretability of the topic structure (Figures 9A–C). Topic activity increased markedly after 2018, in line with the overall expansion of the field (Figure 9D).

Among the seven topics, Topic 0, related to sarcopenia and fibrosis risk; Topic 5, related to lipid metabolism and lifestyle interventions; and Topic 6, related to evolving MAFLD/MASLD terminology and cardiometabolic risk, showed the strongest upward trends and were identified as emerging research directions. By contrast, topics related to basic anthropometric assessment, conventional disease progression, and advanced liver disease with sarcopenia showed slower recent growth. Linear regression analysis confirmed significant upward trends for Topic 5 (R^2^ = 0.679, *p* = 9.75 × 10^−4^), Topic 0 (R^2^ = 0.647, *p* = 5.02 × 10^−3^), and Topic 6 (R^2^ = 0.587, *p* = 4.46 × 10^−2^) (Figures 9E–K; Table 7; Supplementary Table 4). These findings indicate evolving research priorities rather than causal relationships or direct clinical effects.

**Table 7:** BERTopic-derived topics with linear regression trend statistics.

Topic	Linear_r2	Linear_p
0	0.647033558	0.005021504
1	0.412698413	0.085843868
2	0.256553383	0.135182732
3	0.385084597	0.137032374
4	0.2473641	0.143565171
5	0.679399235	0.000975007
6	0.586826347	0.04462312

Linear regression R^2^ and p values are shown for the temporal trend of each topic.

## 4 Discussion

### 4.1 Principal findings and interpretation

This study provides a comprehensive bibliometric and topic-modeling overview of research at the intersection of MASLD and sarcopenia from 2012 to 2025. The findings indicate rapid expansion of the field, accompanied by a shift from descriptive epidemiology toward mechanistic exploration and translational research. Rather than reiterating numerical results, this analysis highlights three major transformations in the research landscape. First, the conceptual framework has evolved from viewing sarcopenia mainly as a late-stage complication of liver disease to recognizing it as a factor potentially associated with disease progression. Second, research attention has shifted from isolated organ pathology to a systemic metabolic perspective, with increasing emphasis on the liver–muscle axis and its integration with cardiometabolic risk. Third, the field appears to be moving toward intervention-oriented strategies, particularly those targeting metabolic regulation, nutrition, and lifestyle modification.

Together, these trends suggest that MASLD–sarcopenia comorbidity is increasingly being conceptualized as a dynamic and potentially bidirectional process embedded within systemic metabolic dysfunction, rather than as a simple coexistence of two conditions.

The three expanding themes identified through BERTopic modeling further reflect shifts in pathophysiological understanding, clinical management priorities, and evolving disease terminology within the MASLD–sarcopenia research field. The growing focus on lipid metabolism dysregulation and lifestyle interventions (Topic 5) may reflect increasing recognition of liver–muscle crosstalk. Lipid metabolic disturbances have been proposed as key links between hepatic and muscular pathologies (23, 24). For instance, free fatty acid–induced lipotoxicity has been associated with hepatocellular injury, skeletal muscle atrophy, and intramuscular fat infiltration, thereby potentially contributing to a self-reinforcing cycle (25, 26). Accordingly, recent studies have moved beyond describing comorbidity toward exploring modifiable metabolic pathways. Lifestyle interventions have also been increasingly investigated for their potential to improve hepatic steatosis while preserving or enhancing muscle mass and overall metabolic health.

Traditionally, sarcopenia has been regarded as a complication of advanced liver disease, with earlier studies focusing on its association with post-transplant outcomes and survival. However, the sustained growth of sarcopenia–fibrosis risk coupling (Topic 0) suggests that research attention has shifted toward earlier disease stages. Emerging evidence indicates that sarcopenia-related parameters may be associated with progression from simple steatosis to steatohepatitis and fibrosis, although the nature of this relationship remains to be fully elucidated (27, 28). As the largest amino acid reservoir and an active endocrine organ, skeletal muscle may influence hepatic inflammatory processes through altered cytokine and myokine signaling.

The increasing prominence of cardiometabolic risk (Topic 6) is consistent with evolving disease definitions that emphasize metabolic dysfunction as a central component. Under this framework, the research scope has expanded beyond liver-specific pathology to include systemic metabolic and cardiovascular outcomes (29). In this context, sarcopenia has been investigated as a potential predictor of cardiovascular risk, with liver and muscle abnormalities jointly contributing to adverse cardiometabolic profiles (30–32). These trends suggest that future studies may increasingly incorporate broader clinical endpoints, including cardiovascular disease, heart failure, and all-cause mortality, rather than focusing solely on liver-related outcomes.

### 4.2 Integration with emerging clinical evidence

To provide clinical context for the bibliometric and topic-modeling findings, we descriptively reviewed five prospective cohort studies from PubMed, including both Asian and Western populations, with follow-up periods ranging from 29 months to 12 years (Table 8). These studies used heterogeneous approaches for sarcopenia or muscle assessment. Specifically, Kim et al., Petermann-Rocha et al., Cho et al., and Choe et al. used bioelectrical impedance analysis (BIA) combined with different adjustment methods, including weight adjustment, BMI normalization, and muscle-to-visceral fat ratio (33–36). In contrast, Hsieh et al. used computed tomography (CT) to quantify skeletal muscle mass and intramuscular fat infiltration (37).

**Table 8:** Integrated summary of five prospective studies from PubMed.

Study	Follow-up	Population	Sarcopenia assessment	MASLD diagnosis	Fibrosis assessment	Key findings	HR (95% CI)
Choe et al.	12 years	Asian population	BIA; BMI-adjusted skeletal muscle mass index	Hepatic steatosis index ≥36	FIB-4 ≥ 2.67	Low muscle mass associated with increased MASLD risk	1.18 (1.11–1.27)
Petermann-Rocha et al.	10 years	European population	BIA; skeletal muscle mass index; grip strength	ICD-10 code K76.0	NR	Low muscle mass and grip strength associated with increased severe MASLD risk	Grip strength: 0.84 (0.80–0.88); Muscle mass: 0.70 (0.66–0.74)
Kim et al.	7.0 years (mean)	Asian population	BIA; weight-adjusted skeletal muscle mass index	Hepatic steatosis index	NR	Higher relative skeletal muscle mass associated with lower MASLD incidence and higher resolution	Incidence: 0.44 (0.38–0.51); Resolution: 2.09 (1.02–4.28)
Cho et al.	3.7 years	Asian population	BIA; skeletal muscle/visceral fat ratio	Ultrasound	FIB-4 ≥ 2.67; NAFLD fibrosis score >0.676	Low muscle/visceral fat ratio associated with higher MASLD and advanced fibrosis risk	MASLD men: 1.92 (1.80–2.05); women: 3.37 (2.99–3.8); fibrosis men: 2.83 (2.19–3.64); women: 7.96 (3.85–16.44)
Hsieh et al.	29 months	Asian population	CT; myosteatosis	Liver biopsy	Transient elastography	Severe myosteatosis associated with increased liver fibrosis progression	>2 kPa: 2.49 (1.15–5.40); ≥7 kPa: 2.09 (1.01–4.34)

Details study design, sample size, muscle-related assessment, MASLD-related outcomes, follow-up duration, and main findings, demonstrating current clinical evidence on MASLD-sarcopenia comorbidity.

Regarding MASLD assessment, most studies relied on imaging modalities, such as hepatic ultrasonography or transient elastography, or standardized scoring systems, such as the Fibrosis-4 index, whereas Hsieh et al. used liver biopsy. Across these studies, lower muscle mass or reduced muscle function was generally associated with higher MASLD incidence or progression-related markers, although the magnitude and direction of associations varied according to study design, exposure definition, and outcome assessment. For instance, Kim et al. reported that relative skeletal muscle mass was inversely associated with MASLD incidence (HR = 0.44) and positively associated with remission probability (HR = 2.09) (33). Petermann-Rocha et al. reported that higher handgrip strength was associated with a lower risk of severe MASLD progression (HR = 0.84 per unit increase), indirectly supporting the relevance of reduced muscle function (34). Cho et al. and Choe et al. similarly described associations between low muscle-to-visceral fat ratio or reduced muscle mass and fibrosis-related risk markers or MASLD risk (HR = 1.18) (35, 36).

Despite differences in assessment tools, population characteristics, and outcome definitions, these studies are broadly consistent with the growing attention to sarcopenia-related measures in MASLD research. Given the limited number of studies, methodological heterogeneity, and lack of formal quality appraisal, these findings should be interpreted cautiously. This review was descriptive in nature and was intended to provide contextual clinical evidence rather than confirm causal relationships. Future studies using standardized muscle assessments and larger prospective cohorts are needed to further clarify these associations.

### 4.3 Biological context for muscle–liver crosstalk

**Figure 10:**
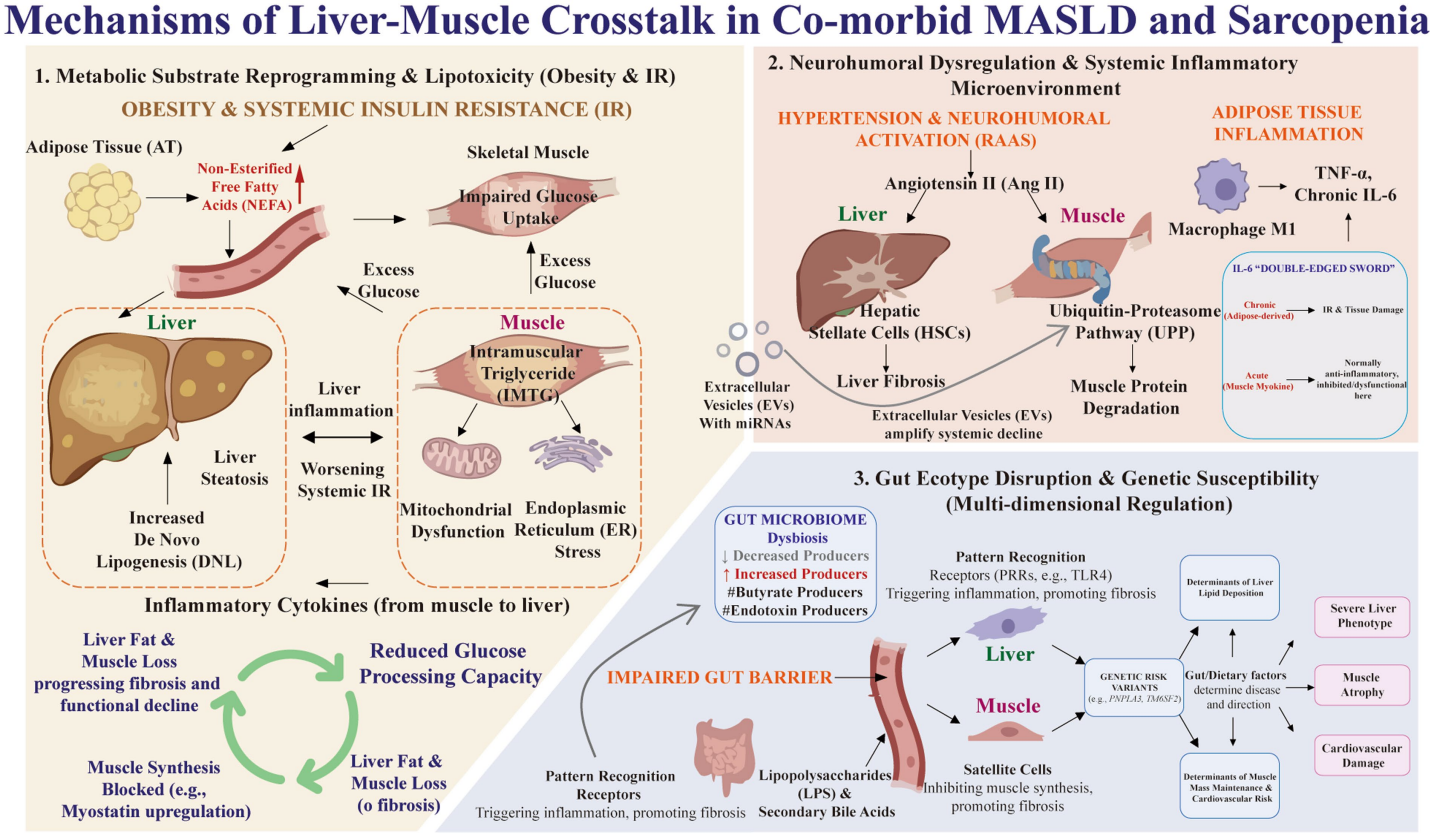
Schematic of liver–muscle crosstalk in MASLD-sarcopenia comorbidity. This diagram delineates three interconnected pathogenic axes: (1) Metabolic substrate reprogramming and lipotoxicity, whereby insulin resistance promotes ectopic hepatic lipid accumulation and intramyocellular fat infiltration; (2) Neurohormonal dysregulation and systemic inflammation, characterized by activation of the renin–angiotensin–aldosterone system (RAAS) and increased production of pro-inflammatory cytokines (e.g., TNF-α, IL-6) derived from M1-polarized macrophages; and (3) Gut microbiota dysbiosis and genetic susceptibility, involving perturbations of the gut–liver–muscle axis alongside key polymorphisms (e.g., *PNPLA3*, *TM6SF2*) that modulate disease progression and phenotypic heterogeneity.

Obesity and insulin resistance are commonly regarded as potential initiating factors in the muscle–liver interaction network, possibly through systemic substrate redistribution and lipotoxicity (13, 38). Under conditions of nutrient excess, impaired insulin signaling may reduce skeletal muscle glucose uptake, thereby redirecting surplus carbohydrates to the liver for *de novo* lipogenesis (DNL) (39, 40). Concurrently, attenuated anti-lipolytic signaling in adipose tissue has been associated with increased release of non-esterified fatty acids (NEFAs) (39). These lipid substrates may accumulate in hepatocytes and skeletal muscle, contributing to steatosis, intramuscular fat deposition, mitochondrial dysfunction, and endoplasmic reticulum stress responses (41, 42). Such metabolic alterations may contribute to a self-reinforcing cycle in which hepatic inflammatory signaling exacerbates systemic insulin resistance, while reduced muscle anabolic capacity further limits glucose utilization. Together, these processes provide a plausible biological context for the interplay between hepatic injury and skeletal muscle wasting (43, 44) (Figure 10).

Hypertension and associated neurohormonal activation have also been proposed as potential links between hepatic and muscular dysfunction. Activation of the renin–angiotensin–aldosterone system (RAAS), particularly increased angiotensin II signaling, has been implicated in hepatic stellate cell activation and muscle protein degradation through the ubiquitin–proteasome pathway (45, 46). In parallel, M1-polarized adipose tissue macrophages secrete proinflammatory cytokines such as TNF-*α* and IL-6, contributing to systemic low-grade inflammation (47, 48). Notably, IL-6 appears to exert context-dependent effects: chronic adipose-derived IL-6 has been associated with insulin resistance and tissue injury, whereas acute muscle-derived IL-6 may have transient anti-inflammatory roles, although these effects may be altered under chronic metabolic stress (49–51). Additionally, extracellular vesicles have been suggested to mediate inter-organ communication by transporting microRNAs and signaling molecules between liver and muscle, potentially amplifying inflammatory responses (52, 53) (Figure 10).

Dysregulation of the gut–liver–muscle axis, together with host genetic background, may also influence disease phenotype and progression. Alterations in gut microbiota composition, including reduced butyrate-producing bacteria and increased endotoxin-producing strains, have been linked to impaired intestinal barrier function and increased translocation of microbial metabolites such as lipopolysaccharide (LPS) (54–56). These metabolites may activate pattern recognition receptors, such as TLR4, thereby triggering downstream inflammatory signaling in hepatic and muscular tissues. Considerable inter-individual variability in response to these factors has been reported. Genetic variants such as PNPLA3 and TM6SF2 have been associated with differences in hepatic lipid accumulation and metabolic risk profiles (36, 57–62). These gene–environment interactions may contribute to variability in disease manifestation, although their precise roles remain to be fully clarified (Figure 10).

In addition to lipotoxicity, inflammation, and gut microbiota dysregulation, mechanistic target of rapamycin (mTOR) signaling has been proposed as a regulatory node linking nutrient status, insulin signaling, and muscle homeostasis. mTOR integrates signals from nutrient availability, growth factors, and cellular energy status and coordinates anabolic and catabolic processes through two major complexes, mTORC1 and mTORC2 (63, 64). In skeletal muscle, mTORC1 is involved in protein synthesis and maintenance of muscle mass, whereas dysregulated mTOR signaling may contribute to impaired muscle anabolism and sarcopenia-related processes (65). In the liver, mTOR signaling has been associated with lipid metabolism and insulin resistance, and nutrient-rich conditions may promote mTORC1-related lipogenic signaling (66, 67).

mTOR signaling may therefore represent a plausible mechanistic bridge between hepatic lipid accumulation, systemic insulin resistance, and skeletal muscle protein turnover (63, 65, 68). However, current evidence regarding its role in MASLD–sarcopenia comorbidity is largely derived from experimental studies. Further clinical and translational research is needed to determine whether mTOR-related pathways can serve as biomarkers or therapeutic targets in this specific comorbidity context (65, 68).

### 4.4 Integrated management and precision nutrition perspectives

The management of MASLD–sarcopenia comorbidity may benefit from integrated strategies that concurrently address fat reduction and muscle preservation (9). Traditional caloric restriction, while effective in reducing hepatic steatosis, may also increase the risk of skeletal muscle loss, highlighting the need for more refined nutritional approaches. These may include adherence to antioxidant-rich dietary patterns, such as the Mediterranean diet, or time-restricted eating regimens, combined with carefully monitored protein intake to support muscle protein synthesis without imposing excessive metabolic burden on the liver (14, 69, 70).

Exercise interventions have also been proposed to shift from purely aerobic training toward combined aerobic–resistance regimens. Aerobic exercise has been associated with improvements in cardiometabolic profiles and reductions in visceral adiposity, whereas resistance training may promote muscle protein synthesis, enhance muscle strength, and improve liver-related parameters (13, 71). The combination of nutritional and exercise strategies may reduce hepatic lipid burden while preserving muscle mass and function, thereby potentially attenuating the reciprocal interaction between metabolic dysregulation and muscle wasting.

In the pharmacological domain, emerging studies have explored strategies to balance metabolic benefits with the preservation of muscle mass. Thyroid hormone receptor *β* agonists, such as resmetirom, have shown liver-targeted metabolic effects in MASH-related clinical studies, while GLP-1 receptor agonists have demonstrated efficacy in promoting MASH resolution (72, 73). However, these agents are often associated with weight reduction, which may increase the risk of muscle loss, particularly in vulnerable populations (74). Consequently, combination therapeutic approaches have been proposed, such as combining GLP-1 receptor agonists with activin receptor antagonists. These strategies are intended to facilitate fat reduction while preserving skeletal muscle mass through modulation of myostatin-related signaling pathways (75–77). Nevertheless, their clinical effectiveness and safety require further validation in large-scale prospective and randomized studies.

Beyond pharmacological approaches, metabolic or bariatric surgery has also been investigated in patients with obesity-related metabolic liver disease. For patients undergoing rapid postoperative weight reduction, perioperative nutritional support and early rehabilitation have been suggested as strategies to mitigate muscle loss. These approaches may help preserve muscle function and support the durability of metabolic improvements over time (77, 78).

Future management may increasingly rely on precision-based and dynamically monitored frameworks. Inter-individual variability in genetic background, gut microbiome composition, and metabolic phenotype suggests that standardized interventions may not be universally effective. Personalized strategies integrating genomic, microbiomic, nutritional, and clinical data may offer potential advantages over conventional approaches. In this context, continuous monitoring of hepatic fat content and skeletal muscle indices could facilitate adaptive adjustments in nutritional and exercise interventions. Such approaches may enhance adherence and support early identification of metabolic and functional decline, particularly in vulnerable populations. However, further prospective studies are required to establish their feasibility, effectiveness, and clinical relevance in real-world settings.

### 4.5 Limitations

Several limitations should be acknowledged. First, this study was restricted to English-language publications, which may introduce language bias and limit the inclusion of region-specific research. Second, discrepancies in indexing and coverage between WOSCC and Scopus may influence the mapping of research networks, and minor inconsistencies may remain despite data harmonization efforts. Third, as a retrospective and descriptive approach, bibliometric analysis primarily maps the landscape of scholarly attention and publication dynamics rather than establishing clinical evidence or causal relationships. The emerging trends identified through topic modeling reflect patterns of academic discourse and should not be interpreted as direct guidance for clinical practice without validation from high-quality studies.

Fourth, the PubMed-based prospective studies were reviewed descriptively, and no formal risk-of-bias assessment or meta-analysis was performed. Therefore, this component should be regarded as contextual evidence rather than confirmatory evidence. Finally, heterogeneity in diagnostic criteria and outcome measures across the included literature may affect the generalizability of the findings. Future research should prioritize methodological standardization and prospective validation to better bridge the gap between research trends and clinical practice.

## 5 Conclusion

Research on MASLD and sarcopenia has evolved from early descriptive associations toward a broader cross-organ framework centered on the liver–muscle axis. Bibliometric and topic-modeling analyses revealed a marked increase in scholarly output over the past decade, together with expanding international collaboration networks. China, South Korea, and the United States emerged as major contributors. The findings suggest that research attention has gradually shifted from descriptive and mechanistic studies toward clinical phenotyping, risk stratification, lifestyle-related interventions, and integrated metabolic management.

Emerging evidence indicates that sarcopenia may represent more than a concomitant phenotype of MASLD and may be associated with disease progression, particularly liver fibrosis. Proposed mechanisms include lipotoxicity, systemic low-grade inflammation, mTOR-related metabolic signaling, and dysregulation of the gut–liver–muscle axis. However, these pathways require further validation through rigorous, hypothesis-driven clinical and mechanistic studies.

Future research should prioritize standardized muscle assessment protocols, further investigation of inter-organ metabolic crosstalk, and prospective validation of multi-target nutrition, exercise, and metabolic intervention strategies. By mapping the evolution of scholarly attention, this study provides a framework for understanding the MASLD–sarcopenia research landscape and may support the transition from single-organ approaches toward more comprehensive management of systemic metabolic dysfunction.

## Data Availability

The original contributions presented in the study are included in the article/Supplementary material, further inquiries can be directed to the corresponding author.

## Supplementary material

SUPPLEMENTARY TABLE S1 Search strategies employed in WOSCC, Scopus, and PubMed.

SUPPLEMENTARY TABLE S2 Average citations per publication over time. Trend of mean citations per publication in WOSCC, Scopus, and merged datasets by publication year.

SUPPLEMENTARY TABLE S3 Top 30 keywords and their frequencies.

SUPPLEMENTARY TABLE S4 BERTopic-derived topics with corresponding literature counts.

SUPPLEMENTARY FIGURE S1 Global overview and bibliometric analysis of the datasets. **(A)** Global overview of the WOSCC dataset from 2012 to 2025. **(B)** Global overview of the Scopus dataset from 2012 to 2025. **(C)** Integrated overview of the deduplicated WOSCC–Scopus dataset from 2012 to 2025. The panels display key bibliometric indicators, including timespan, number of sources, total documents, annual growth rate, number of authors, single-authored documents, international co-authorship percentage, co-authors per document, authors’ keywords, total references, document average age, and average citations per document.

SUPPLEMENTARY FIGURE S2 Dual-map overlay analysis of journals. This visualization illustrates citation relationships across academic disciplines. The left map represents citing journals, indicating where the research was published, and the right map represents cited journals, indicating the foundational literature referenced by these publications. The colored curves trace citation flows between fields. Two main citation trajectories originating from the Medicine/Medical/Clinical cluster are highlighted: one toward Health/Nursing/Medicine and another toward Molecular/Biology/Genetics. These pathways underscore the interdisciplinary nature of the field, showing how clinical and medical studies integrate knowledge from health sciences and molecular biology.

SUPPLEMENTARY FIGURE S3 Top 30 references with the strongest citation bursts. This figure presents the results of citation burst detection analysis, identifying references that experienced rapid increases in citations during specific periods. For each of the top 30 references, the figure provides citation details, publication year, burst strength, and the start and end years of the burst period. The timeline from 2012 to 2025 illustrates the temporal dynamics of scholarly influence, with red segments marking periods of intense citation activity.
