# Supplementary material for: MASLD and sarcopenia research (2012–2025): a multi-database bibliometric analysis
Source: Front Nutr. 2026 Jun 12;13:1834112. doi: 10.3389/fnut.2026.1834112 (PMC13305728; doi:10.3389/fnut.2026.1834112)
Supplement: SUPPLEMENTARY TABLE S1 — Search strategies employed in WOSCC, Scopus, and PubMed. [file Table_1.docx]

| **Database** | **Search Strategy** | **Search Date** | **Filtering Conditions** |
| --- | --- | --- | --- |
| WOSCC | TS1 = ("Non-alcoholic Fatty Liver Disease" OR "Nonalcoholic Fatty Liver Disease" OR "Metabolic Associated Fatty Liver Disease" OR "Metabolic-associated Fatty Liver Disease" OR "Metabolic Dysfunction-associated Steatotic Liver Disease" OR "Metabolic Dysfunction Associated Steatotic Liver Disease" OR "Metabolic Steatohepatitis" OR "Nonalcoholic Steatohepatitis" OR "Non-alcoholic Steatohepatitis" OR "NAFLD" OR "MAFLD" OR "MASLD" OR "MASH" OR "NASH");  TS2 = (sarcopen* OR myopeni* OR dynaponi*);  TS=TS1 AND TS2 | The literature search was conducted on February 10, 2026. | English-language publications from January 1, 2012, to December 31, 2025, including Articles and Review Articles. |
| Scopus | ( TITLE-ABS-KEY ( "Non-alcoholic Fatty Liver Disease" OR "Nonalcoholic Fatty Liver Disease" OR "Metabolic Associated Fatty Liver Disease" OR "Metabolic-associated Fatty Liver Disease" OR "Metabolic Dysfunction-associated Steatotic Liver Disease" OR "Metabolic Dysfunction Associated Steatotic Liver Disease" OR "Metabolic Steatohepatitis" OR "Nonalcoholic Steatohepatitis" OR "Non-alcoholic Steatohepatitis" OR "NAFLD" OR "MAFLD" OR "MASLD" OR "MASH" OR "NASH" ) AND TITLE-ABS-KEY ( sarcopen* OR myopeni* OR dynaponi* ) ) | The literature search was conducted on February 10, 2026. | English-language publications from January 1, 2012, to December 31, 2025, including Articles and Review Articles. |
| PubMed | ("Non-alcoholic Fatty Liver Disease"[Mesh] OR MASLD[Title/Abstract] OR NAFLD[Title/Abstract] OR MAFLD[Title/Abstract]) AND ("Sarcopenia"[Mesh] OR "Sarcopenia"[Title/Abstract]) | The literature search was conducted on February 10, 2026. | Prospective cohort studies that satisfied the eligibility criteria |
