# Supplementary material for: MASLD and sarcopenia research (2012–2025): a multi-database bibliometric analysis
Source: Front Nutr. 2026 Jun 12;13:1834112. doi: 10.3389/fnut.2026.1834112 (PMC13305728; doi:10.3389/fnut.2026.1834112)
Supplement: SUPPLEMENTARY TABLE S2 — Average citations per publication over time. Trend of mean citations per publication in WOSCC, Scopus, and merged datasets by publication year. [file Table_2.docx]

| **Year** | **WOSCC** | **Scopus** | **WOSCC+Scopus** |
| --- | --- | --- | --- |
| 2012 | 1,026.50 | 1,143.50 | 1,143.50 |
| 2013 | 44.67 | 55.50 | 60.80 |
| 2014 | 220.00 | 160.25 | 146.80 |
| 2015 | 87.75 | 82.60 | 82.60 |
| 2016 | 118.73 | 131.50 | 131.58 |
| 2017 | 88.45 | 182.33 | 150.87 |
| 2018 | 71.89 | 62.83 | 70.00 |
| 2019 | 70.05 | 84.31 | 75.06 |
| 2020 | 47.42 | 51.70 | 47.08 |
| 2021 | 33.48 | 55.39 | 53.22 |
| 2022 | 21.75 | 30.74 | 27.68 |
| 2023 | 15.95 | 26.97 | 24.21 |
| 2024 | 10.19 | 25.70 | 22.19 |
| 2025 | 6.25 | 6.55 | 6.09 |
