# Supplementary material for: MASLD and sarcopenia research (2012–2025): a multi-database bibliometric analysis
Source: Front Nutr. 2026 Jun 12;13:1834112. doi: 10.3389/fnut.2026.1834112 (PMC13305728; doi:10.3389/fnut.2026.1834112)
Supplement: SUPPLEMENTARY TABLE S3 — Top 30 keywords and their frequencies. [file Table_3.docx]

| **Keywords** | **Count** |
| --- | --- |
| sarcopenia | 335 |
| non-alcoholic fatty liver disease | 284 |
| insulin resistance | 214 |
| obesity | 202 |
| prevalence | 140 |
| nonalcoholic steatohepatitis | 118 |
| nonalcoholic fatty liver | 115 |
| skeletal muscle | 112 |
| fibrosis | 99 |
| risk | 96 |
| metabolic syndrome | 94 |
| liver cirrhosis | 89 |
| liver fibrosis | 86 |
| mortality | 82 |
| fatty liver disease | 81 |
| sarcopenic obesity | 80 |
| muscle mass | 75 |
| fatty liver | 74 |
| body composition | 71 |
| risk factor | 66 |
| liver transplantation | 65 |
| body mass | 59 |
| complication | 59 |
| inflammation | 57 |
| hepatic steatosis | 56 |
| steatohepatitis | 56 |
| diabetes mellitus | 55 |
| cardiovascular disease | 54 |
| major clinical study | 52 |
| diagnosis | 49 |
