# Supplementary material for: MASLD and sarcopenia research (2012–2025): a multi-database bibliometric analysis
Source: Front Nutr. 2026 Jun 12;13:1834112. doi: 10.3389/fnut.2026.1834112 (PMC13305728; doi:10.3389/fnut.2026.1834112)
Supplement: SUPPLEMENTARY TABLE S4 — BERTopic-derived topics with corresponding literature counts. [file Table_4.docx]

| **Topic** | **Count** | **Label** | **Representation** |
| --- | --- | --- | --- |
| 0 | 140 | Sarcopenia and Fibrosis Risk in NAFLD Patients | ['sarcopenia', 'nafld', 'patients', 'muscle', 'liver', 'mass', 'risk', 'associated', 'fibrosis', 'ci'] |
| 1 | 72 | Body Composition and Muscle Strength in NAFLD | ['muscle', 'nafld', 'mass', 'liver', 'body', 'fat', 'strength', 'fibrosis', 'index', 'low'] |
| 2 | 70 | Metabolic Risk and Disease Progression in NAFLD/NASH | ['nafld', 'liver', 'disease', 'fatty', 'nonalcoholic', 'patients', 'risk', 'nash', 'associated', 'metabolic'] |
| 3 | 50 | MASLD and Myosteatosis in Advanced Liver Disease | ['masld', 'liver', 'metabolic', 'disease', 'patients', 'cirrhosis', 'steatotic', 'dysfunctionassociated', 'myosteatosis', 'studies'] |
| 4 | 46 | Frailty and Post-Transplant Outcomes in Liver Disease | ['frailty', 'patients', 'liver', 'lt', 'transplantation', 'cirrhosis', 'mortality', 'transplant', 'outcomes', 'associated'] |
| 5 | 43 | Obesity, Lipid Metabolism and Lifestyle Interventions | ['obesity', 'metabolic', 'disease', 'liver', 'lipid', 'weight', 'exercise', 'diseases', 'fatty', 'muscle'] |
| 6 | 39 | MAFLD and Cardiometabolic Risk (ASCVD) | ['mafld', 'liver', 'disease', 'fatty', 'risk', 'patients', 'metabolic', 'clinical', 'steatosis', 'ascvd'] |
