# Supplementary figures and images for: MASLD and sarcopenia research (2012–2025): a multi-database bibliometric analysis
Source: Front Nutr. 2026 Jun 12;13:1834112. doi: 10.3389/fnut.2026.1834112 (PMC13305728; doi:10.3389/fnut.2026.1834112)

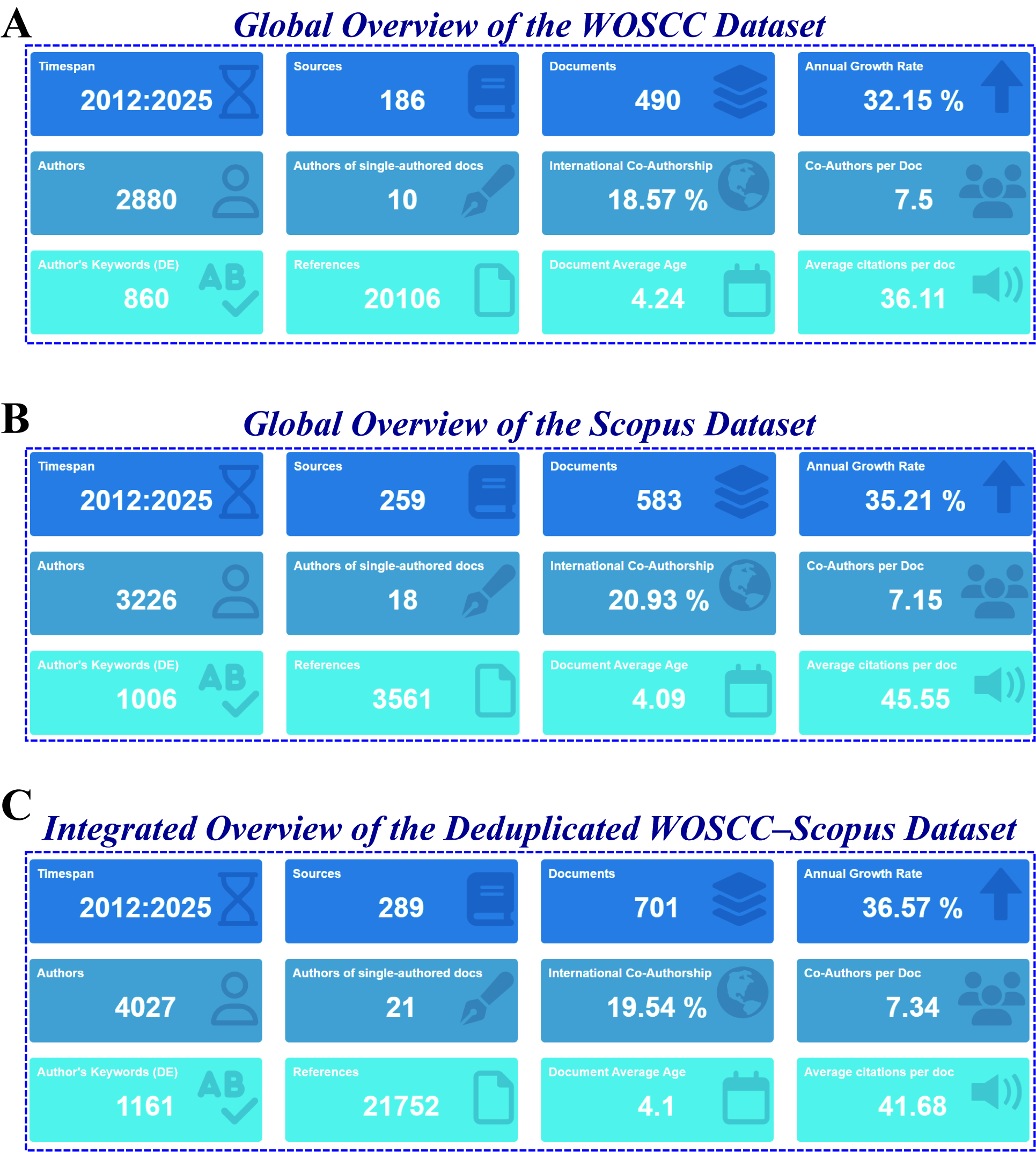

Supplement: SUPPLEMENTARY FIGURE S1 — Global overview and bibliometric analysis of the datasets. (A) Global overview of the WOSCC dataset from 2012 to 2025. (B) Global overview of the Scopus dataset from 2012 to 2025. (C) Integrated overview of the deduplicated WOSCC–Scopus dataset from 2012 to 2025. The panels display key bibliometric indicators, including timespan, number of sources, total documents, annual growth rate, number of authors, single-authored documents, international co-authorship percentage, co-authors per document, authors’ keywords, total references, document average age, and average citations per document. [file Image_1.tif]

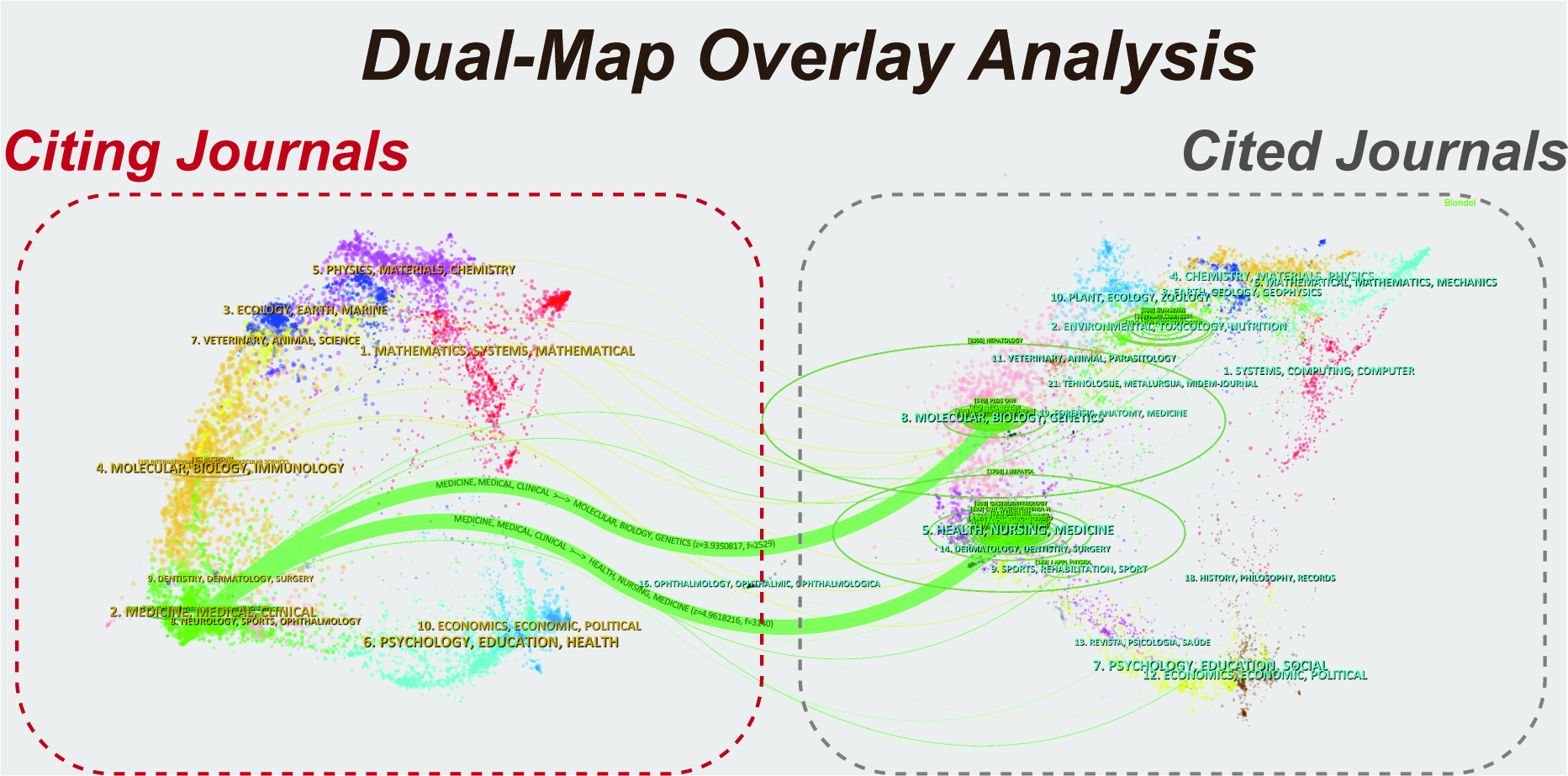

Supplement: SUPPLEMENTARY FIGURE S2 — Dual-map overlay analysis of journals. This visualization illustrates citation relationships across academic disciplines. The left map represents citing journals, indicating where the research was published, and the right map represents cited journals, indicating the foundational literature referenced by these publications. The colored curves trace citation flows between fields. Two main citation trajectories originating from the Medicine/Medical/Clinical cluster are highlighted: one toward Health/Nursing/Medicine and another toward Molecular/Biology/Genetics. These pathways underscore the interdisciplinary nature of the field, showing how clinical and medical studies integrate knowledge from health sciences and molecular biology. [file Image_2.tif]

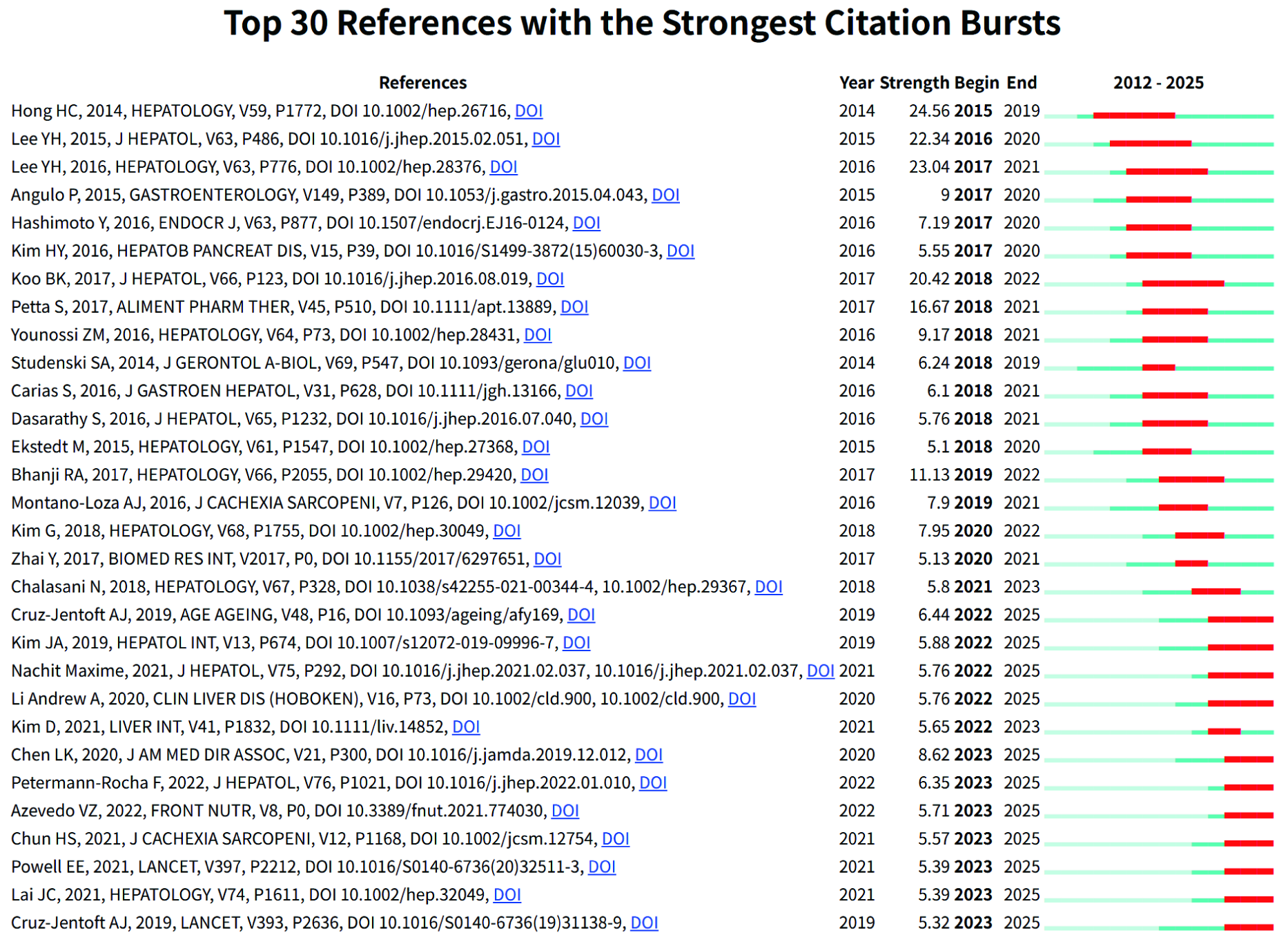

Supplement: SUPPLEMENTARY FIGURE S3 — Top 30 references with the strongest citation bursts. This figure presents the results of citation burst detection analysis, identifying references that experienced rapid increases in citations during specific periods. For each of the top 30 references, the figure provides citation details, publication year, burst strength, and the start and end years of the burst period. The timeline from 2012 to 2025 illustrates the temporal dynamics of scholarly influence, with red segments marking periods of intense citation activity. [file Image_3.tif]
